# Nickel Ferrite Nanoparticles for In Vivo Multimodal Magnetic Resonance and Magnetic Particle Imaging

**DOI:** 10.1021/acsanm.5c03013

**Published:** 2025-07-16

**Authors:** Vít Herynek, Lenka Rajsiglová, Michal Babič, Monika Švábová, Jaroslav Kohout, Miroslav Veverka, Tomáš Kmječ, Lenka Kubíčková, Jiří Karela, Filip Gregar, Martin Loula, Stanislava Matějková, Luděk Šefc, Luca Vannucci

**Affiliations:** † Center for Advanced Preclinical Imaging (CAPI), First Faculty of Medicine, 37740 Charles University, Prague 120 00, Czech Republic; ‡ Laboratory of Immunotherapy, Institute of Microbiology, 86879 Czech Academy of Sciences, Prague 142 00, Czech Republic; § Polymer and Colloid Immunotherapeutics, Institute of Macromolecular Chemistry, Czech Academy of Sciences, Prague 162 00, Czech Republic; ∥ Department of Low Temperature Physics, Faculty of Mathematics and Physics, 37740 Charles University, Prague 180 00, Czech Republic; ⊥ Department of Magnetics and Superconductors, Institute of Physics, Czech Academy of Sciences, Prague 162 00, Czech Republic; # Department of Analytical Chemistry, Faculty of Science, 98735 Palacký University Olomouc, Olomouc 771 46, Czech Republic; ∇ Analytical Laboratory, Institute of Organic Chemistry and Biochemistry, Czech Academy of Sciences, Prague 160 00, Czech Republic

**Keywords:** nickel ferrite nanoparticles, saturation magnetization, magnetic particle imaging, r_2_ relaxivity, magnetic resonance imaging

## Abstract

Magnetic nanoparticles have been at the center of biomedical research for decades, primarily for their applications in magnetic resonance imaging (MRI) and magnetic particle imaging (MPI). Superparamagnetic particles, typically based on iron oxide crystals, are effective in both modalities, although each requires distinct magnetic properties for optimal performance. We investigated the performance of nanoparticles based on a nickel-substituted ferrite core and compared them to standard maghemite iron oxide nanoparticles. We synthesized γ-Fe_2_O_3_ and Ni_*x*_Fe_2–*x*_O_3_ nanoparticles and coated them with a statistical copolymer poly­(*N*,*N*-dimethylacrylamide-*co*-acrylic acid). In vitro testing included X-ray diffraction (XRD), Mössbauer spectroscopy, magnetometry, magnetic resonance relaxometry, magnetic particle spectroscopy, and imaging. In vivo testing involved monitoring of nanoparticle biodistribution using MPI and MRI after intracardial application in a murine model. Mössbauer spectra suggest that the Ni-substituted nanoparticles consist of a stoichiometric NiFe_2_O_4_ ferrite and a poorly crystalline antiferromagnetic iron­(III) oxide-hydroxide phase. Amorphous-like impurities in Ni_*x*_Fe_2–*x*_O_3_ nanoparticles were probably responsible for lower saturation magnetization than that of γ-Fe_2_O_3_ nanoparticles, as was proved by magnetometry, which led to lower *r*_2_ relaxivity. However, MPI revealed a higher signal in the spectrum and superior imaging performance of Ni_*x*_Fe_2–*x*_O_3_ compared to γ-Fe_2_O_3_ particles, likely due to shorter Néél and Brownian relaxation times. Both types of nanoparticles showed similar performance in bimodal MRI/MPI imaging in vivo. They were detected in the liver immediately after application and appeared in the spleen within 24 h. Long-term localization in the lymph nodes was also observed. Substituting an iron with a nickel ion in the core altered the magnetic properties, leading to lower saturation magnetization and an increased signal in the magnetic particle spectra, which enhanced their performance in MPI. This study demonstrates that γ-Fe_2_O_3_ and Ni_*x*_Fe_2–*x*_O_3_ nanoparticles are both suitable for combined MRI/MPI imaging; magnetic particle imaging provides a highly specific signal for anatomical magnetic resonance images.

## Graphical abstract

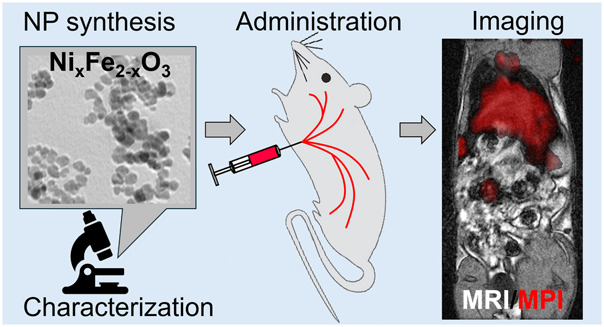

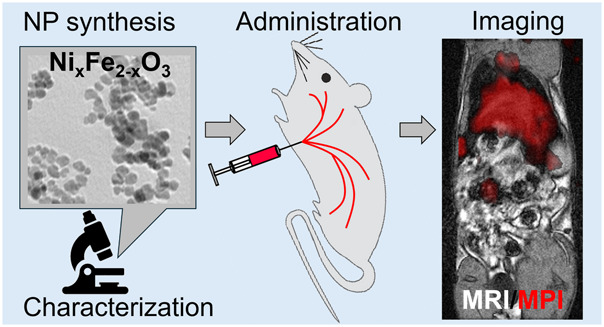

## Introduction

Magnetic nanoparticles have already been used in both medical research and clinical practice. They serve as efficient contrast agents, can be conjugated with therapeutic molecules, enable magnetic targeting, or act as a medium for thermal therapy.^1^ Although the use of iron oxide nanoparticles in clinical magnetic resonance imaging (MRI) still raises questions regarding adverse effects, benefits, and risks,^2^ which led to discontinuation of some of them,^3,4^ we are convinced that magnetic nanoparticles still have great potential as contrast agents not only for MRI, but also for magnetic particle imaging (MPI), especially in preclinical research.^5^

The effect of magnetic nanoparticles on the MRI signal is well-understood. They generate local magnetic field inhomogeneities that alter the Larmor frequency of ^1^H spins in their surroundings. This results in a loss of phase coherence of precessing ^1^H spins, leading to a faster decay of the transverse component of magnetization and shortening of transversal relaxation time *T*_2_ and *T*_2_*.^6^ This decay is described by the transverse relaxation rate *R*_2_ = 1/*T*_2_. Magnetic nanoparticles, characterized by the so-called relaxivity (*r*_2_ = (1/*T*_2_–1/*T*_2w_)/*c*, where *T*_2w_ is the relaxation time of water and *c* is the molar concentration) thus cause hypointensities in *T*_2_ weighted or *T*_2_* weighted magnetic resonance (MR) images.

In contrast to MRI, where the signal originates from hydrogen nuclei and nanoparticles modify their signal, MPI directly detects the magnetic nanoparticles.^7^ MPI does not provide anatomical information, and images representing nanoparticle distribution need to be colocalized with anatomical images, such as MRI or CT. MPI images are generated by the interaction of superparamagnetic nanoparticles with an external magnetic field. This field is a superposition of a selection field (SF) and oscillating drive fields (DF). The nanoparticles’ magnetic moments follow the oscillating drive field, generating a measurable signal. The selection field is a static gradient field with an area with zero magnetic field (so-called field-free point (FFP) in the case of a single point with zero field, or field-free line (FFL) in the case of a line in the space with zero field). The high gradient of the selection field saturates magnetic moments outside the field-free region (FFP or FFL) and its close vicinity, so the detected signal originates only from the unsaturated field-free region, enabling signal localization. Imaging is achieved by moving the FFP or FFL across the sample while recording the signal of magnetic moments that respond to the oscillating magnetic field.

Ideally, the magnetic moments of superparamagnetic nanoparticles with fast relaxation follow the oscillating field according to the Langevin function, which is nearly linear at low field intensities and nonlinear near the saturation. These nonlinearities produce higher harmonics in the recorded signal. Filtering of the excitation frequency isolates the signal from the sample, and the higher harmonics are used to reconstruct the image.

Although MPI’s basic principles are straightforward, the magnetization dynamics in aqueous nanoparticle dispersions is complicated due to relaxation processes,^8,9^ which may not be sufficiently fast. Two key relaxation mechanisms should be considered for nonimmobilized particles: the Néel relaxation (magnetic moment rotation within the crystal structure) and Brownian relaxation (rotation of the entire particle). Additionally, the steepness of the magnetization curve and magnetic field strength required to reach saturation (or nonlinear zone) are critical, often more than the absolute value of saturation magnetization.

Brownian relaxation depends predominantly on nanoparticle size and coating, which mediates interactions with the solvent. In contrast, Néel relaxation and magnetic properties such as magnetization and saturation, depend strongly on the core composition. Even among iron oxides, magnetic properties vary with structural differences. Iron oxides include multiple forms, such as iron­(II) oxide, wüstite (FeO),^10^ magnetite (Fe_3_O_4_),^11^ iron­(III) oxide (Fe_2_O_3_), alpha phase, hematite (α-Fe_2_O_3_),^12^ beta phase, (β-Fe_2_O_3_),^13^ gamma phase, maghemite (γ-Fe_2_O_3_),^14^ epsilon phase (ε- Fe_2_O_3_),^15^ each with distinct magnetic properties, which depend on the method of synthesis.^16^ Magnetite (Fe_3_O_4_) finds its place in material science, energy storage, environmental applications, and in biomedicine as a probe for diagnostics and therapy.^17^ Maghemite (γ-Fe_2_O_3_) is considered a crucial material for various applications including nanomedicine and biosensors, but it is also used in spin electronic devices, high-density magnetic recording etc.^18^ It has a modified spinel structure schematized by (Fe)­[Fe_5/3_ □_1/3_]­O_4_, where () denotes the tetrahedral A-sites, [] denotes the octahedral B-sites, and □ represents the vacancies.^19^

Substituting iron ions in the crystal structure with other metal ions introduces further variations and leads to new properties, which may enhance nanoparticle performance for specific applications. For example, manganese ferrites can achieve saturation magnetization values between 19.6 and 52 Am^2^kg^–1^.^20^ Manganese–zinc ferrites known for their ferrimagnetic or superparamagnetic properties^21,22^ exhibit excellent *r*_2_ relaxivity, making them effective MRI contrast agents.^23–25^ High magnetization leading to high *r*_2_ relaxivity was found also in cobalt–zinc ferrites, moreover, its value is adjustable by change of Zn content.^26^ Interestingly, magnetic particle spectroscopy revealed that Néel relaxation is the dominant mechanism of the effective relaxation time in zinc ferrites, while Brownian relaxation dominates in the case of cobalt ferrites due to their magnetically blocked state at room temperature.^27^ While iron-based magnetic nanoparticles are mostly used as contrast agents for MRI, the introduction of other metallic ions may also substantially change their biochemical properties, and they may serve also as theranostic or multifunctional therapeutic agents. Substituted Mn–Zn iron oxides were found to be suitable for magnetic hyperthermia, tunable by the ratio of manganese and zinc.^28,29^ Ni-substituted particles were reported to exhibit differential cytotoxicity to cancer cell lines.^30^

In this study, we explored the effect of substituting an iron ion in a ferrite core with nickel in nanoparticles, characterized them, and investigated influence of substitution on MR relaxivity and magnetic particle spectra. Finally, the particles were tested as probes in MPI, both in vitro and in vivo using an animal model. MPI images were colocalized with MRI, where the nanoparticles serve as an unspecific contrast agent.

## Methods

Figure 1 shows a schematic sketch of the whole experiment. Each step is described in detail below.

**Figure 1:**
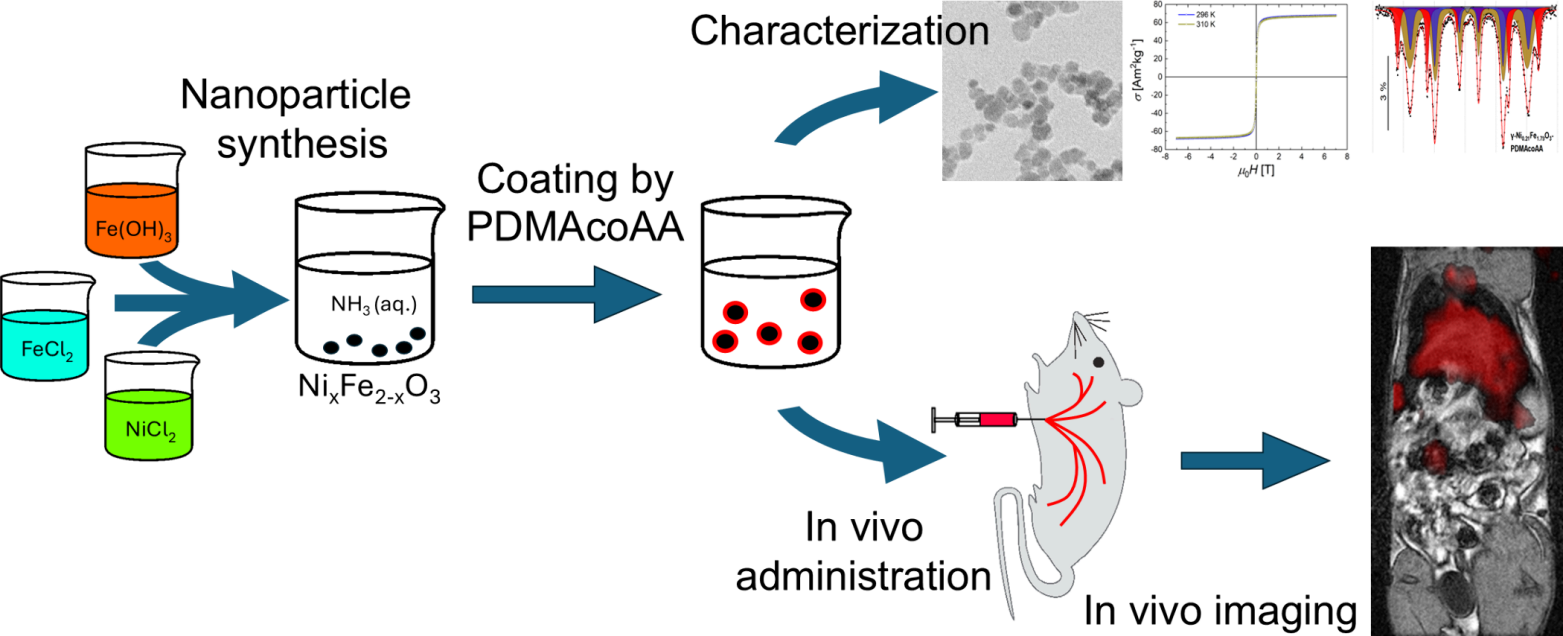
Schematic sketch of the experiment. It included synthesis of γ-Fe_2_O_3_ and nickel-substituted Ni_*x*_Fe_2–*x*_O_3_ nanoparticles, their coating by a statistical copolymer of *N*,*N*-dimethyl acrylamide and acrylic acid, characterization by various physical and chemical methods, and in vivo testing, which included administration to experimental animals and noninvasive imaging of nanoparticle biodistribution using MRI and MPI.

## Particle Preparation

Two types (γ-Fe_2_O_3_ and nickel-substituted Ni_*x*_Fe_2–*x*_O_3_) of nanoparticles were investigated. Both types of nanoparticles were prepared according to the previously published method of preparation of ferrite nanoparticles.^31^ In the case of Ni-containing nanoparticles, the modification of the original method consisted of replacing 50% of the molar amount of FeCl_2_ with NiCl_2_. Aqueous solutions of FeCl_3_·6H_2_O, FeCl_2_·4H_2_O (and NiCl_2_·6H_2_O) (Sigma-Aldrich, Prague, Czech Republic) were prepared by dissolving salts in water and then purified by MCE membrane (22 μm) filtration prior to use. The solution of FeCl_3_ (0.2M, 100 mL, was treated with aqueous ammonia (0.5M, 100 mL, Sigma-Aldrich) under sonication for 2 min. Then the FeCl_2_ solution (0.2M, 50 mL), or the mixture of FeCl_2_ and NiCl_2_ solutions (0.2M, 25 + 25 mL) was added. The dispersion was poured into aqueous ammonia (0.5M, 250 mL) and stirred. Black particles, presumably of magnetite nature, were formed. The mixture was stirred for 1 h and allowed to sediment. The supernatant was discarded and the sedimented particles were purified by repeated addition of water followed by magnetic separation and decantation five times. The trisodium citrate dihydrate solution (0.1M, 12 mL, Sigma-Aldrich) and the NaClO solution (5%, 19 mL) were added to the colloid and sonicated for 10 min. Consequently, the colloids were repeatedly washed with ultrapure water (18.2 MΩ) via magnetic separation until a spontaneous peptization (approximately 15–20 cycles) was achieved. Finally, the colloid was transferred to a clean laminar box, sonicated for 5 min, and filtered through a 0.45 μm sterile PVDF syringe filter and its concentration was measured gravimetrically from 2 × 0.5 mL colloid samples.

The expected ratio of nickel and iron in Ni_*x*_Fe_2–*x*_O_3_ based on the ratio of substrates used for the synthesis, and similarity of both samples was Ni:Fe = 1:5, i.e., the corresponding formula would be Ni_0.33_Fe_1.67_O_3_.

The synthesis scheme was as follows:

$$4\mathrm{Fe}(\mathrm{OH}{)}_{3}+{\mathrm{FeCl}}_{2}+{\mathrm{NiCl}}_{2}\to \left(\mathrm{FeO}\cdot \mathrm{NiO}\right)\cdot 2\left({\mathrm{Fe}}_{2}O_{3}\right)\overset{\mathrm{NaCIO}}{\to }3\left({\mathrm{Ni}}_{0.33}{\mathrm{Fe}}_{1.67}O_{3}\right)$$

## Coating of Nanoparticles

The coating of the nanoparticles was done by a statistical copolymer of *N*,*N*-dimethyl acrylamide (DMA) (90 wt %) and acrylic acid (AA) (10 wt %). The copolymer (PDMAcoAA) was prepared by aqueous solution free radical polymerization: 0.9 g of DMA and 0.1 g of AA (monomers were purified of the MEHQ stabilizer by passing over 4 cm high column of basic alumina) was dissolved in ultrapure water (18.2 MΩ) and 0.01 g of potassium persulfate (initiator) was added. The mixture was purged with Argon (10 min), sealed in a glass flask, and polymerized at 70 °C for 8 h. After polymerization, the polymer was purified by dialysis against water using a 14 kDaA cutoff cellulose membrane. The resulting polymer was *M*_*n*_ ∼ 150 kDa, *Đ* ∼ 1.8 (measured by size exclusion chromatography with multiangle light scattering detector). For the coating of the nanoparticles, we used 5 mL of water (18.2 MΩ) solution containing 44 mg of PDMAcoAA, which was added under sonication (10% of 200W ultrasonic horn with a 1 mm tip) to an aliquot of stock colloid of magnetic particles containing 44 mg of nanoparticles (dry mass) diluted by water to achieve 10 mL of the final colloid. The polymer solution was sterilized by passing through a 0.22 μm sterile PVDF syringe filter; the coating procedure was performed in a clean laminar box.

## Inductively Coupled Plasma Optical Emission Spectrometry (ICP-OES)

Fe and Ni in the nanoparticle samples of γ-Fe_2_O_3_ and Ni_*x*_Fe_2–*x*_O_3_ (both coated and uncoated) were determined using inductively coupled plasma optical emission spectrometer with a radial view of the plasma (Arcos MV, Spectro, Kleve, Germany). Calibration of the spectrometer was performed using diluted multielement standard solution (Analytika s r.o., Prague, Czech Republic). Multiple emission lines were observed for both elements, each line was carefully checked for interferences prior to its evaluation. The calibration was performed in concentration range 0–5 mg/L, the detection limit was from to 0.6 μg/L for Fe and from 2 μg/L for Ni (in the analyzed solution).

Sample preparation: Prior preparation, samples were homogenized using a shaker and an ultrasonic bath. Approximately 50 μL of a sample (precisely weighed) was pipetted into a glass tube. Then 550 μL of HCl and subsequently 550 μL HNO_3_ (both Analpure grade, Analytika s r.o., Prague, Czech Republic) were added, and 50 μL of yttrium (serving as an internal standard for the analysis) standard solution (Analytika s r.o., Prague, Czech Republic) were added before adjusting the sample volume to 25 mL with deionized water, final amount precisely weighed. The analysis was performed in a total of three replicates.

## Dynamic Light Scattering (DLS)

The coating efficiency, colloidal stability, and hydrodynamic size of the initial and polymer coated particles in the aqueous suspension were examined by dynamic light scattering (DLS), measuring the hydrodynamic diameter (*D*_h_), the polydispersity index (PDI) and the zeta potential (ζ) (Zetasizer Nano Series ZEN3500, Malvern, Worcestershire, UK). All values were measured for diluted aqueous dispersions of particles in disposable folded DTS1070 capillary cells. The values in Table 1 are averages of 5 consecutive measurements.

**Table 1:** Average Nanoparticle Size (*D*_*n*_) and Size Dispersity (*Đ*) Determined from TEM Images, Hydrodynamic Size (*D*_h_), Polydispersity Index (PDI), and Zeta Potential (ζ) of Bare and Coated γ-Fe_2_O_3_ and Ni_*x*_Fe_2–*x*_O_3_ Nanoparticles Measured by DLS^a^

	*D*_*n*_ (nm)	*Đ*	*D*_h_ (nm)	PDI	ζ (mV)	pH
γ-Fe_2_O_3_ bare	9.4	1.32	75.2 ± 0.2	0.14 ± 0.01	–64 ± 2	7.6
γ-Fe_2_O_3_–PDMAcoAA	10.3	1.30	169 ± 2	0.16 ± 0.02	–37.9 ± 0.7	4.9
Ni_*x*_Fe_2–*x*_O_3_bare	9.8	1.32	58.2 ± 0.5	0.151 ± 0 0.004	–50 ± 2	7.3
Ni_*x*_Fe_2–*x*_O_3_–PDMAcoAA	10.6	1.27	122 ± 1	0.189 ± 0 0.006	–23.8 ± 0.2	5.9

a The variable pH reflects differences in the protolytic activity of the nanoparticle surfaces. Sizes derived from TEM are reported without standard deviations, as their distributions are better characterized by dispersity (*Đ*). Errors in the DLS measurements represent the standard deviations from five consecutive measurements, reflecting the repeatability of the measurement. However, these do not provide information about the hydrodynamic size distribution, which is instead described by the polydispersity index (PDI).

## Transmission Electron Microscopy (TEM)

The shape, average diameter, and size distribution of both uncoated and coated particles were evaluated directly from microphotographs obtained by a transmission electron microscope (FEI-TEM, Tecnai G2 Spirit, Oregon, USA). Microphotographs were analyzed with ImageJ analysis software^32^ using at least 1000 particles. The sizes of particles were measured manually from at least 8 different images of one sample. The average diameter (*D*_*n*_) and dispersity (*Đ*) were calculated according to the following formulas:

$$D_{n}=\frac{\sum n_{i}D_{i}}{\sum n_{i}}$$ 1

$$Đ=\frac{D_{w}}{D_{n}};<1,\infty )$$ 2

where *n*_*i*_ is the number of the nanoparticles, *D*_*i*_ is the diameter of the nanoparticles, and *D*_*w*_ is the weight-average diameter of the nanoparticles calculated as

$$D_{w}=\frac{\underset{i}{\sum }n_{i}D_{i}^{4}}{\underset{i}{\sum }n_{i}D_{i}^{3}}$$ 3

## Magnetometry

Magnetization measurements of bare and PDMAcoAA-coated γ-Fe_2_O_3_ and Ni_*x*_Fe_2–*x*_O_3_nanoparticle samples were performed in both aqueous suspensions and dried samples. Aqueous suspensions were placed in a short glass cuvette sealed with a Teflon stopper; the cuvette was then fitted into a plastic straw and measured using a superconducting quantum interference device (SQUID) magnetometer MPMS XL (Quantum Design, USA). Dry samples were prepared by drying the aqueous suspensions in a desiccator using a vacuum pump. The samples were placed in plastic cuvettes and measurement was performed using a vibrating sample magnetometer PPMS9 (Quantum Design, USA). The magnetization isotherms of the samples were measured at 296 K (23 °C - room temperature) and 310 K (37 °C - body temperature) in the magnetic field range of −7 to 7 T. The saturation magnetization σ_*s*_ value of the samples was obtained by fitting the magnetization data σ­(*H*) in the range of field μ_0_ *H* from 2 to 7 T by the law of approach to saturation magnetization in the form

$$\sigma (H)=\sigma_{s}(1-a/H-b/H^{2})+\chi H$$ 4

where *H* is the intensity of the applied magnetic field, *a*, *b*, χ are constants.^33^

To investigate subtle differences at low magnetic field amplitudes relevant for MPI, the magnetization curves of PDMAcoAA-coated nanoparticles were fitted by the Langevin function in a form

$$\sigma =\sigma_{s}L(x)=\sigma_{s}(\coth(x)-1/(x))$$ 5

where σ_*s*_ = *n*μ*, x* = μμ_0_ *H/k*_B_ *T*, μ is the mean magnetic moment of nanoparticles, μ_0_ is the permeability of vacuum, *n* is the number of nanoparticles per unit volume, *k*_B_ is the Bolzmann constant, and *T* is the absolute (thermodynamic) temperature. The derivatives of the magnetization curves in the range of ±14 mT were compared. Analysis of magnetization curves was performed according to the Supporting Information of a study published by Poryvai et al.^34^

## Mösbauer Spectroscopy

The ^57^Fe Mössbauer spectra of the pure γ-Fe_2_O_3_ and Ni_–_substituted nanoparticles were collected in transmission geometry using a constant-acceleration spectrometer equipped with a ^57^Co/Rh source at room temperature. The calibration of the spectrometer and the values of isomer shifts are given with respect to a room-temperature Mössbauer spectrum of an α-Fe foil. The Mössbauer spectra at liquid helium temperature and in an external magnetic field of *B*_ext_ = 6 T, oriented perpendicularly to the γ-ray direction, were acquired in a Janis bath cryostat. Spectra were evaluated using the Confit (version no. 4.10.10.1)^35^ and MossWin 4.0 fitting programmes.^36^

## X-ray Diffraction (XRD)

The powder X-ray diffraction (XRD) was employed to determine phase purity, crystal structure and size of crystallites of bare samples. The room-temperature diffractograms were recorded in the Bragg–Brentano geometry on a Bruker D8 Advance diffractometer, using Cu K_α_ radiation. The patterns were analyzed in FULLPROF^37^ by the Rietveld method, employing the Thompson-Cox- Hastings pseudo-Voigt profile to resolve strain and size contributions to the line broadening. The instrumental profile was determined based on a strain-free LaB_6_ standard.

## In Vitro MR Relaxometry

The stock suspensions were diluted to 0.0703, 0.1405, and 0.281 mM Fe for the case of γ-Fe_2_O_3_ particles (coated and uncoated), and 0.0698, 0.1396, and 0.2793 mM of metallic ions in the case of Ni_*x*_Fe_2–*x*_O_3_ (coated and uncoated) particles.

The *T*_2_ relaxation times of 1 mL aliquots were measured using a MiniSpec mq20 relaxometer (Bruker, Ettlingen, Germany) working at a 0.47 T magnetic field at two temperatures (room temperature 23 °C, and body temperature 37 °C). Each aliquot was measured three times using a standard CPMG sequence (echospacing 2 ms, repetition time TR = 5 s, 1 dummy scan, and 4 acquisitions). The *T*_2_ relaxation times were converted to relaxivities *r*_2_ according to a formula

$$r_{2}=\frac{\frac{1}{T_{2}}-\frac{1}{T_{2w}}}{c}$$ 6

where *T*_2*w*_ is the *T*_2_ relaxation time of the solvent (water) and *c* is the molar concentration of metallic ions.

The samples were mixed and treated in an ultrasonic bath before each measurement.

The results obtained from the repetitive measurements and different concentrations were then averaged. Uncoated particles tended to aggregate during repetitive measurements, namely at higher temperature. Repetitions apparently laden with the error caused by aggregation were excluded before averaging.

## In Vitro Magnetic Particle Spectroscopy (MPS) and Imaging (MPI)

Both MPS and MPI were performed using a field-free point MPI scanner (Bruker BioSpin MRI GmbH, Ettlingen, Germany) with focus field coils (16 mT) and a mouse Rx coil.

For in vitro measurements, 8 μL samples of suspensions of all tested nanoparticles (concentration 4.4 mg/mL) were inserted into special calibration test tubes 2 × 2 × 2 mm^3^. Spectra were obtained after excitation in one direction only, drive field DF = 14 mT, number of acquisitions AC = 128. The signal was processed by ParaVision software (Bruker BioSpin MRI GmbH, Ettlingen, Germany).

To evaluate imaging quality, the same samples were measured using a modified protocol described in our previous study,^19^ which consisted of measurement of a low-matrix calibration (matrix 5 × 5 × 3, field of view FOV = 10 × 10 × 6 mm^3^, 2 mm isotropic resolution, DF = 14 mT, SF = 2.5 T/m, number of acquisitions NA = 256, frequency range 60–1250 kHz); the cubic sample matched exactly the voxel size. Three calibration measurements (providing three system functions) were performed for each sample. The sample was then moved to seven different positions within the FOV and the sample was scanned three times in each position. The images were evaluated with all three system functions, i.e., we obtained 9 images for each position.

Two image parameters were evaluated: the signal-to-noise ratio (S/N - the signal from the voxel containing the sample divided by the average signal from all other voxels) and the signal dispersion (the signal in a vicinity of the sample divided by the signal in the voxel with the sample). Parameters were evaluated by an in-house script ISNER (written in Matlab, MathWorks, Natick, MA, USA). The average values and standard deviations from the three measurements, three independent evaluations with different system functions, and seven positions were calculated.

## In Vivo Imaging

To evaluate the biodistribution and half-life of nanoparticles in living organisms, we used preclinical imaging methods MRI and MPI. Coated nanoparticles γ-Fe_2_O_3_–PDMAcoAA and Ni_*x*_Fe_2–*x*_O_3_–PDMAcoAA were applied to healthy Balb/c mice purchased from AnLab (Prague, Czech Republic). The animals were kept in individually ventilated cages (12:12 h light–dark cycle, 22 ± 1 °C, 60 ± 5% humidity). The study used adult female mice (8 weeks old at the beginning of the experiment) with free access to water and a standard rodent diet. The experiments were approved by the Laboratory Animal Care and Use Committee of the First Faculty of Medicine, Charles University, and the Ministry of Education, Youth and Sports of the Czech Republic (MSMT-2309/2018–4). The approved protocol was in accordance with the Act of the Czech Parliament for the Protection of Animals Against Cruelty No. 246/1992 and the Directive 2010/63/EU of the European Parliament. The experiments were designed on the principle of the ‘Three Rs’ (replacement, reduction, and refinement).

The animals were subjected to nanoparticle application and scanning by MPI and MRI for three months after application.

Nanoparticle application:

8- to 10-week-old female Balb/c mice (5 animals per group) were put under general anesthesia by intraperitoneal administration of a 300 μL ketamine-xylazine mixture (10 mg/mL of ketamine and 2 mg/mL of xylazine). Animals were kept on a heated pad and injected intracardially with 50 μL of either γ-Fe_2_O_3_–PDMAcoAA or Ni_*x*_Fe_2–*x*_O_3_–PDMAcoAA nanoparticles (9 mg/kg).

Both MPI and MRI were performed under general anesthesia induced and maintained by spontaneous breathing of isoflurane in air (3% for induction, 1–2% for maintenance). Vital functions were controlled during the measurement. The mice were placed in a special holder that fits both into MPI and MRI scanners in an exactly defined position.

MRI measurements were performed using a 1 T MRI scanner ICON (Bruker BioSpin MRI GmbH, Ettlingen, Germany) equipped with a mouse whole-body solenoid coil. The MRI protocol consisted of a localizer, a fast gradient echo sequence (echo time TE = 3 ms, repetition time TR = 107.27 ms, flip angle FA = 30°, number of acquisitions AC = 2, matrix 128 × 128, field of view FOV = 60 × 60 mm^2^, slice thickness 1.5 mm) in three orthogonal directions for orientation; a FLASH sequence with mild *T*_1_/*T*_2_* weighting (TE = 4, 160 ms, FA = 80°, AC = 128, matrix 256 × 128, FOV = 51.2 × 25.6 mm^2^, slice thickness 1 mm) in coronal direction; and a FLASH sequence with strong *T*_2_* weighting (TE = 8, 400 ms, FA = 60°, AC = 8, and the same geometry). The intensity of the signal was evaluated in the liver, spleen, renal cortex, and renal medulla. As the intensity of the MR signal strongly depends on instrument settings, the values were related to the signal in the femoral muscle, because we do not expect significant deposition of the nanoparticles in the muscle tissue.

To cover the whole mouse body, patch scanning with an MPI patch sequence implemented by Bruker was used in the case of MPI. Patch system function measurement was performed using the same samples as were used for in vitro MPS and MPI with the following parameters: DF = 14 × 14 × 14 mT, SF = 1.25 T/m in the *x* and *y* directions, 2.5 T/m in the *z* direction, matrix size 24 × 24 × 12, 1 mm spatial resolution.

Together with a single-patch measurement, two patch patterns were used:

a) Two patches in the *x* direction, 1 patch in the *y* and *z* directions with 50% patch overlap and AC = 128. The final FOV was 36 × 24 × 12 mm^3^; the pattern covered most of the chest and abdomen of the mouse. Scanning time: 5 s.
b) Three patches in the *x* direction, 2 patches in the *y* direction, and 3 patches in the *z* direction with 50% patch overlap, AC = 8. This pattern covered the entire mouse (without the tail). The final FOV covered by the multipatch sequence was 48 × 36 × 24 mm^3^. The total scanning time was 3 s.

Individual patches were reconstructed using ParaVision software (Bruker BioSpin MRI GmbH, Ettlingen, Germany).

The file containing the sequence of data from the individual patches was loaded into VOMMPI software,^38^ which merged the patches into one 3D image. Overlapping parts were averaged with the use of Gaussian weighting and suppression of the border slices. Finally, the coronal MPI images were interpolated to the same matrix as the MRI images, and 8 central slices were summed and colocalized with the MRI images using ImageJ software.^32^

## Postmortem Nickel Quantification

Another three mice (female Balb/c purchased from AnLab, Prague, Czech Republic) were used for the quantification of Ni (originating from Ni_*x*_Fe_2–*x*_O_3_ nanoparticles). Two volumes (either 50 or 150 μL, equivalent to 8.8 mg/kg and 26.4 mg/kg) of Ni_*x*_Fe_2–*x*_O_3_–PDMAcoAA suspension (4.4 mg/mL) were administered intracardially (as in the previous experiment). The third animal served as a control. The mice were sacrificed after 24 h by anesthesia overdose with subsequent cervical dislocation, organs (the spleen, liver, kidney, mesenteric lymph nodes, lung, and brain) were extracted and the nickel content was determined by a modified procedure previously published by Kuba et al.^39^ Briefly, organ and tumor samples (wet weight) were digested using nitric acid and hydrogen peroxide in the Milestone MLS 1200 Mega microwave digestion system (Milestone, Italy). The total amount of Ni was determined by inductively coupled plasma mass spectrometry (ICP-MS) using Agilent 7700x ORS-ICP-MS (Agilent Technologies, Santa Clara, CA, USA) with an external 10-point calibration within 0.5 to 10 000 μg/L. The modified ICP-MS method was verified by analysis of the matrix matched standard reference material (SRM) 1566b Oyster tissue. The verification of the ICP-MS method covered the evaluation of the limit of detection (LOD) and the limit of quantification (LOQ), linearity, trueness, and precision.

## Results and Discussion

The precipitation method for nanoparticle synthesis was selected based on our previous experience and a comprehensive evaluation of its outcomes in terms of morphology, magnetic properties, and subsequent handling. In terms of morphology, the chosen precipitation method yielded particles with a very narrow size distribution. Regarding magnetic properties, we were able to synthesize nanoparticles with saturation magnetization values reaching approximately 90% of that of bulk maghemite, indicating a well-ordered crystalline structure with minimal defects and without “non-magnetic” amorphous phases.^40^ Unlike solvothermal or thermal decomposition methods, the precipitation method does not require any surface-active agents, which is advantageous for constructing particle shells and ensures better stability and biocompatibility in biological environments. In contrast, solvothermal or thermal decomposition methods would require a complex ligand-exchange process for the adsorbed surfactant. Furthermore, coprecipitation synthesis employs less toxic chemicals and is easily scalable.

The tested nanoparticles were subjected to analytical and physical characterization. ICP-OES was used to determine the precise iron-to-nickel atomic ratio. As expected, the Ni content in γ-Fe_2_O_3_ particles (both coated and uncoated) was below detection limit. The w­(Fe)/w­(Ni) ratio in Ni_*x*_Fe_2–*x*_O_3_ was, within experimental error, essentially the same for both uncoated (7.94 ± 0.07) and PDMAcoAA coated particles (8.00 ± 0.04. This ratio corresponds to a core composition of Ni_0.21_Fe_1.79_O_3_. The stochiometric coefficient of oxygen is based on the assumption of maghemite formation, as reported in previous studies,^41,42^ where the original γ-Fe_2_O_3_ was doped with nickel­(II) cation. However, due to experimental limitations, the precise oxygen content is difficult to determine, and the actual stoichiometric coefficient of oxygen may slightly differ from 3.

TEM revealed nanoparticle shape and size, which was *D*_*n*_ = 9.38 nm (and dispersity *Đ* = 1.32) for uncoated γ-Fe_2_O_3_, and *D*_n_ = 9.81 nm (*Đ* = 1.32) for uncoated Ni_*x*_Fe_2–*x*_O_3_. The coated nanoparticles were slightly bigger, γ-Fe_2_O_3_–PDMAcoAA had diameter *D*_*n*_ = 10.33 nm (*Đ* = 1.3) and Ni_*x*_Fe_2–*x*_O_3_–PDMAcoAA had diameter *D*_n_ = 10.59 nm (*Đ* = 1.27), see Figure 2 and Table 1. There is no substantial difference in dispersity *Đ*, which supports the hypothesis, that the mechanism of particle formation is independent of Ni doping, and that the cores did not erode by the coating procedure.

**Figure 2:**
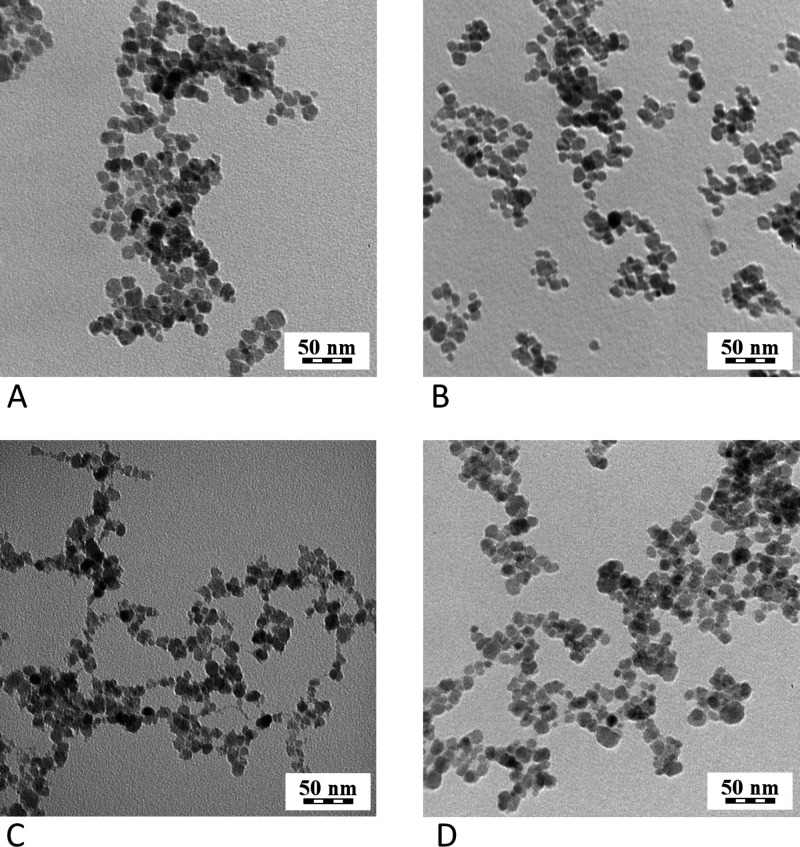
TEM micrograph of uncoated γ-Fe_2_O_3_ (A), coated γ-Fe_2_O_3_–PDMAcoAA (B), uncoated Ni_*x*_Fe_2–*x*_O_3_ (C), and coated Ni_*x*_Fe_2–*x*_O_3_–PDMAcoAA (D) nanoparticles.

The hydrodynamic size, polydispersity index, and zeta potential of both bare and coated nanoparticles are listed in Table 1. Not surprisingly, the hydrodynamic size of coated particles is much larger than that of bare ones. It is caused by two main effects: measurement comprehends the presence of a hydration shell including the coating and the result is calculated by different statistics. (In the case of DLS diameter, the statistical mean is the *Z* average, which is in analogy with eq 3) proportional to the ratio *D*_*Z*_ ∼ *D* ^5^/*D* ^4^. This *Z* average is more sensitive to bigger entities in comparison with statistical means of lower order.) The difference in *D*_h_ of the bare nanoparticles can be explained by a different level of agglomeration due to a different level of salinity in the supernatant, which can be supported by different ζ, but the values of PDI do not support that. A more probable explanation can be found in different volumes of hydration shells. The different hydrodynamic size of coated γ-Fe_2_O_3_–PDMAcoAA compared to Ni_*x*_Fe_2–*x*_O_3_– PDMAcoAA is also interesting, because their sizes determined from TEM images were similar. It can be caused by particle agglomeration or by inhomogeneous redistribution of coating polymer (*Z* average is more sensitive to larger particles). The aggregation of the particle cores can be caused by two phenomena: (i) a drop in ζ below the level of stability (30 mV in absolute value), which could be the case of Ni_*x*_Fe_2–*x*_O_3_–PDMAcoAA or by (ii) bridging with polymer chains. However, the insignificant changes in the values of PDI do not support the explanation by agglomeration or coating inhomogeneity. The ratios of the *D*_h_ values R = coated/uncoated are 2.25 (γ-Fe_2_O_3_) and 2.1 (Ni_*x*_Fe_2–*x*_O_3_), what indicates an almost identical increase in the hydrodynamic volume of the particles by their coating.

The XRD patterns (Figure 3) of both bare samples showed a single crystalline phase with the cubic spinel structure of the *Fd*3̅*m* symmetry, implying that the vacancies in octahedral sites are randomly distributed. The lattice parameters of γ-Fe_2_O_3_ and Ni_0.21_Fe_1.79_O_3_ were refined to *a* = 8.3520(8) and 8.364(2) Å. The mean crystallite sizes (volume-weighted), derived from the line broadening, were determined to be *D*_XRD_ ∼8 and ∼11 nm.

**Figure 3:**
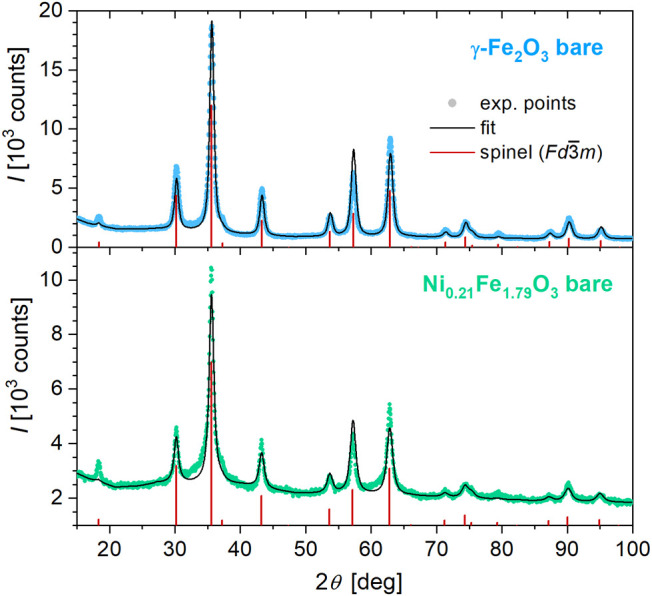
Powder X-ray diffractogram of the bare γ-Fe_2_O_3_ (top) and Ni-substituted Ni*_x_* Fe_2–_ *_x_* O_3_ (Ni_0.21_Fe_1.79_O_3_) nanoparticles. The black line shows the fit by the Rietfeld method, while red vertical lines mark the refined reflection positions of the spinel structure.

The zero-field Mössbauer spectra (see Figure S6 in Supporting Information) showed an asymmetric sextet, whose decomposition into two magnetic components, attributed to the tetrahedral and octahedral Fe sites, remained ambiguous. In contrast, the application of an external magnetic field *B*_ext_ = 6 T clearly splits the spectra into two well-resolved magnetic components (see Figure 4), allowing for discrimination between Fe^3+^ ions whose spins are oriented parallel to the external magnetic field and those oriented antiparallel, as follows from the ferrimagnetic arrangement. The results of the fit are summarized in Table S1 in Supporting Information. The coating of the nanoparticles with PDMAcoAA does not result in any significant modification of the spectra.

**Figure 4:**
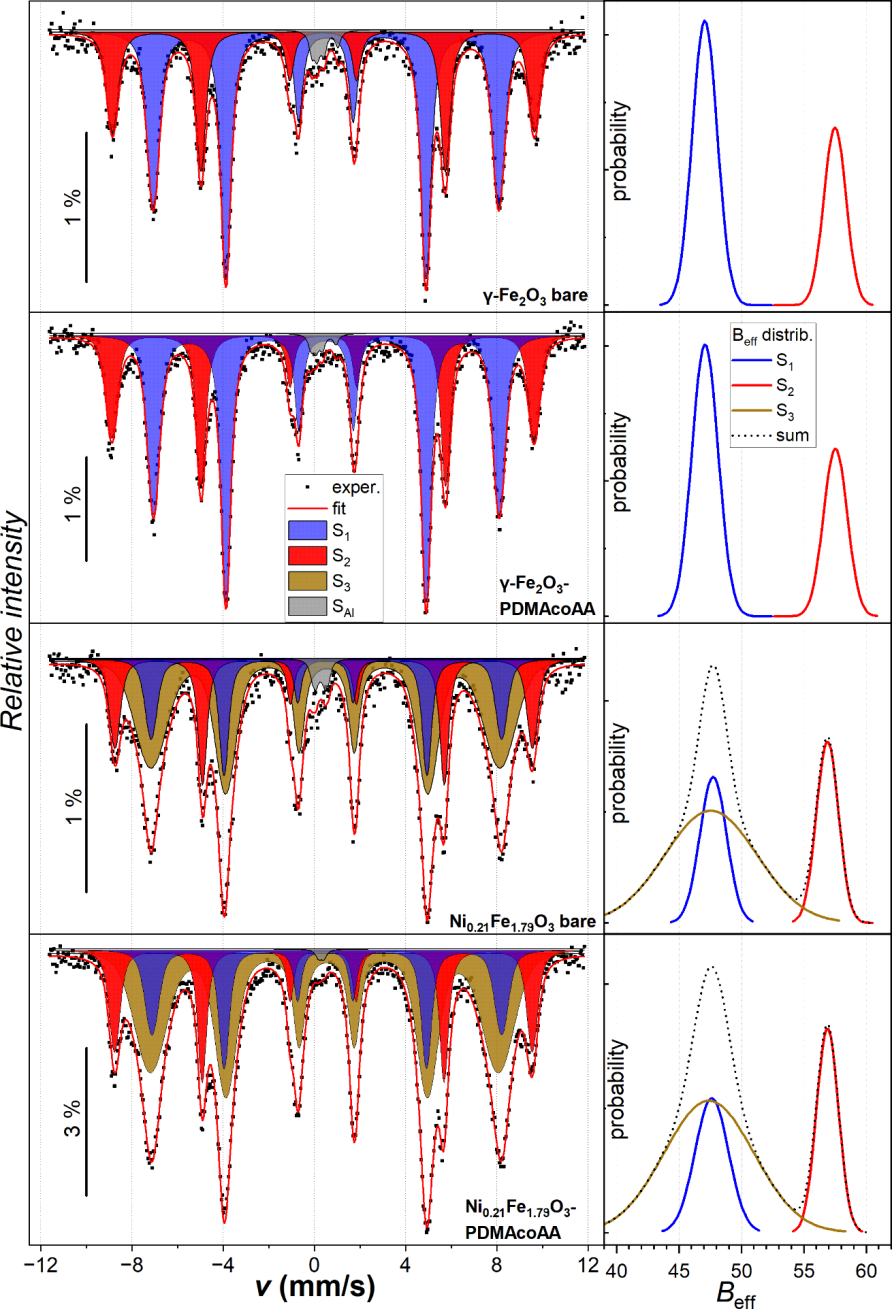
Mössbauer spectra of bare and PDMAcoAA-functionalized γ-Fe_2_O_3_ and Ni-substituted nanoparticles at 4.2 K in the external magnetic field *B*_ext_ = 6 T; the distributions of the effective hyperfine field are shown to the right of the corresponding spectrum (the blue and red colors represent the octahedral and tetrahedral sites, respectively, the brown line represents FeOOH).

The observed isomer shifts for all samples at liquid helium temperature, *IS*_T_ = 0.38 ± 0.02 mm/s in tetrahedral and *IS*_O_ = 0.50 ± 0.02 mm/s in octahedral sites, are in good agreement with those reported for Fe^3+^ cations in nanosized γ-Fe_2_O_3_ particles, specifically *IS*_T_ = 0.36 ± 0.02 mm/s and *IS*_O_ = 0.47 ± 0.03 mm/s,^43^ or *IS*_T_ = 0.38 ± 0.03 mm/s and *IS*_O_ = 0.50 ± 0.02 mm/s.^19^ Nearly zero quadrupole shift *QS* (i.e., the mutual shift of the center of outer lines of the sextet and the center of the remaining four lines) of both sextets corresponds to the cubic structure of maghemite.

In both bare and PDMAcoAA-coated γ-Fe_2_O_3_ nanoparticles, the mean effective magnetic field on ^57^Fe nuclei reaches *B*_eff_ = 57.5 ± 0.3 T in tetrahedral and *B*_eff_ = 47.0 ± 0.3 T in octahedral sites in an applied field of *B*_ext_ = 6 T. These values are consistent with reported ranges *B*_eff_ = 56.4–57.9 T and *B*_eff_ = 46.9–47.6 T, respectively, in dependence on the particle sizes of maghemite.^43^ The distribution of iron between the two sites, indicated by the ratio of integral intensities of the corresponding spectral components *I*_T_/ *I*_O_ = 0.56 ± 0.02, slightly differs from the theoretical distribution in bulk maghemite, which provides *I*_T_/ *I*_O_ = 0.6 for a cubic crystal structure with the *Fd*3̅*m* space group with a random distribution of vacancies.^44^ This deviation may indicate the presence of an amorphous-like impurity with Fe^3+^ in the octahedral environment, most probably FeOOH. The signal of such impurity overlaps significantly with the contribution from octahedral sites in the cubic structure of maghemite, leading to an apparent overestimation of their relative area. Considering the ideal stoichiometry of γ-Fe_2_O_3,_ the content of these impurities would amount to ∼ 4–5 at% Fe. Although their presence is not unambiguously detectable in XRD, it can be deduced from the broadened baseline of the diffraction lines, complicating the Rietveld refinement.

No other Fe_2_O_3_ polymorphs,^45^ namely α-Fe_2_O_3_ (hematite), β-Fe_2_O_3_, ε-Fe_2_O_3_, or other iron-containing crystalline impurities, such as magnetite (Fe^3+^)­[Fe^2+^Fe^3+^]­O_4_ ^46^ or ferrite with Fe^2+^ ions in octahedral sites in the cubic structure,^47^ were detected in Mössbauer spectra within the accuracy of the measurement, which was 1–2 at%. The observed D_Al_ doublet in the spectra corresponds to the aluminum foil in which the samples were measured to achieve better thermal contact (see Figure S6 in Supporting Information).

The spectra of the Ni-substituted samples reveal three components: S_1_ and S_2_ sextets corresponding to the octahedral and tetrahedral sites in the *Fd*3̅*m* crystal structure, and a broad S_3_ sextet (see Figure S6 and Table S1 in Supporting Information). The change of the effective hyperfine fields of S_1_ and S_2_ in the applied field aligns with the ferrimagnetic order of the spinel phase, i.e., *B*_eff_ increases by ∼ 6 T in the tetrahedral site, while it decreases for the octahedral site by roughly the same value. In contrast, S_3_ only broadens and the mean *B*_eff_ almost does not change, which points either to an antiferromagnetic order or a random orientation of magnetic moments even in the applied field. Hyperfine parameters of the S_3_ sextet correspond to Fe^3+^ in FeOOH.^48^ While it is difficult to deduce the content of any amorphous-like impurity due to the unknown distribution of Ni ions among the individual sites in the *Fd*3̅*m* crystal structure, as well as the unknown distribution of Fe and Ni ions in the impurity, its presence can be inferred also from the XRD data. Assuming that all Ni is present in the spinel phase and does not form oxide-hydroxide, nickel remains in the highly stable Ni^2+^ (in contrast to Ni^3+^) and occupies preferentially octahedral sites in the *Fd*3̅*m* structure. Then we can deduce that the samples consist of a practically stoichiometric (Fe^3+^)­[Fe^3+^Ni^2+^]­O_4_ phase (hyperfine parameters similar to nickel ferrite particles e.g. in^49^ and a poorly crystalline antiferromagnetic iron­(III) oxide-hydroxide phase. At the same time, no Fe^2+^ signal was observed in the Mössbauer spectra.

Magnetometry revealed the superparamagnetic behavior of both γ-Fe_2_O_3_ and Ni_*x*_Fe_2–*x*_O_3_ nanoparticles at measured temperatures. Magnetization curves are shown in Figure 5A,B. The saturation magnetization of Ni_*x*_Fe_2–*x*_O_3_ nanoparticles was substantially lower than that of γFe_2_O_3_, see Table 2. The coated nanoparticles revealed lower magnetization in the dry state, which was caused by the fact that it was related to the total mass, which is higher by the contribution of the coating. Lower saturation magnetization of nickel substituted maghemite nanoparticles has already been described in literature,^50,51^ it was reported in the range 25 to 40 Am^2^/kg depending on the crystallinity and particle size, which strongly affects magnetic properties of the nanoparticles. Reduced saturation magnetization has been found also in magnetite nanoparticles doped with nickel; saturation magnetization varied with the amount of nickel in the core.^52^

**Figure 5:**
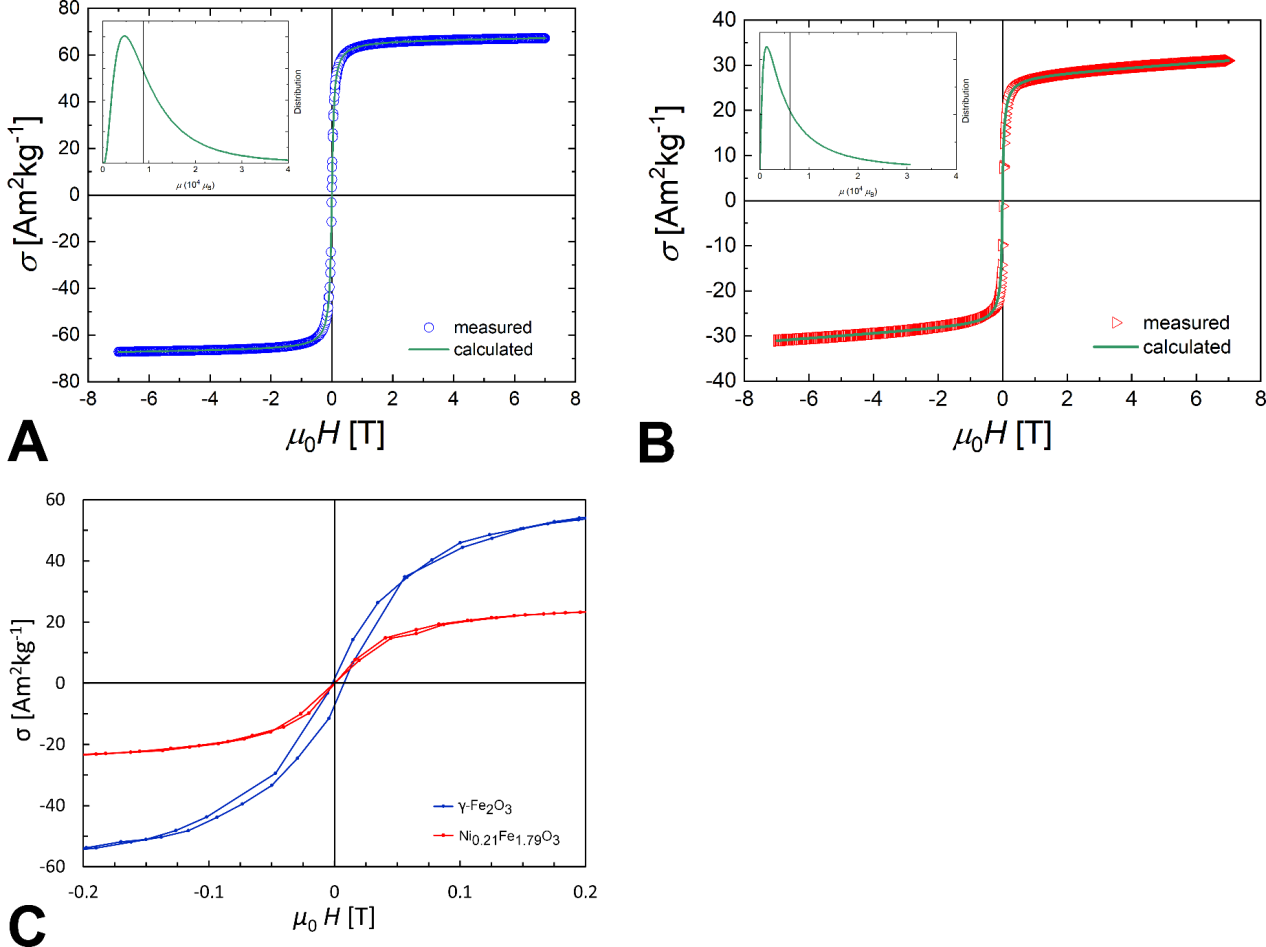
Magnetization isotherms of dried samples of bare γ-Fe_2_O_3_ (A) and Ni_0.21_Fe_1.79_O_3_ (B) nanoparticles measured at body (37 °C) temperature. A comparison of both magnetization curves at low fields is shown in (C). The insets show the distribution of magnetic moments.

**Table 2:** Mass Saturation Magnetization at 23°C and 37°C of the Tested Nanoparticles in Water Suspension and Dried Samples

	mass saturation magnetization (Am^2^/kg)			
	23 °C watersusp.	37 °C water susp.	23 °C dried	37 °Cdried
γ-Fe_2_O_3_ bare	63.0 ± 1.9	64.1 ± 0.6	67.76 ± 0.05	66.80 ± 0.08
γ-Fe_2_O_3_ - PDMAcoAA	66.4 ± 0.5	65.9 ± 1.5	36.44 ± 0.03^a^	35.79 ± 0.01^a^
Ni_*x*_Fe_2–*x*_O_3_ bare	30.7 ± 1.4	30.4 ± 1.0	30.23 ± 0.02	29.49 ± 0.01
Ni_*x*_Fe_2–*x*_O_3_–PDMAcoAA	27.3 ± 0.5	26.6 ± 0.6	15.98 ± 0.01^a^	15.61 ± 0.02^a^

a The weight of the PDMAcoAA coating is not subtracted from the weight of the dried magnetic nanoparticles.

Examination of the magnetization at low field (Figure 5C) revealed slight hysteresis in the case of γFe_2_O_3._ This may indicate longer Néél relaxation time and consequently worse performance in magnetic particle spectroscopy and imaging.

In addition, the doping by Ni can also affect the structure and composition (in the sense of hydroxylation) of crystallites’ surface, which may result in variations in volume of hydration shells in colloidal state, as mentioned in the explanation of hydrodynamic size measurements.

Fitting of the magnetization curves with the Langevin function (eq 5) revealed a substantial difference in term σ_*s*_, which corresponds to saturation magnetization, however, mean value of magnetic moment of nanoparticles μ*,* which defines the curve linearity at low fields (±14 mT, which corresponds to MPI relevant drive field range), was (within the error of the fitting procedure) the same for both γ-Fe_2_O_3_ and Ni_*x*_Fe_2–*x*_O_3_ particles. The first derivative (which determines the curve steepness) differed, but its relative change (responsible for nonlinear behavior) was the same for both γ-Fe_2_O_3_–PDMAcoAA and Ni_*x*_Fe_2–*x*_O_3_–PDMAcoAA particles, which implicates a similar contribution to the magnetic particle signal at higher harmonics. Therefore, we do not expect any influence of the different saturation magnetization on nanoparticle performance in MPI. For a more descriptive explanation and plotted derivatives, see the Supporting Information and Figures S1 and S2.

Interestingly, the distribution of magnetic moments (see the insets in Figure 5A,B) differed for the two types of nanoparticles. The mean value of the magnetic moment was lower for Ni_*x*_Fe_2–*x*_O_3_. This behavior could be caused by different nanoparticle sizes; however, this would contradict the results of TEM. Therefore, we speculate that during nickel oxidation, a thin magnetically disordered surface layer was formed on the surface of nickel-substituted nanoparticles, causing a decrease in the mean magnetic moment.

Although XRD results are inconclusive regarding the ratio and precise nature of the spinel phase and the amorphous-like impurity in the Ni-doped samples, Mössbauer spectra suggest that the samples consist of a practically stoichiometric NiFe_2_O_4_ ferrite and a poorly crystalline antiferromagnetic iron­(III) oxide-hydroxide phase. Such impurity, whether in a paramagnetic or antiferromagnetic state, can significantly reduce the value of magnetization of Ni-substituted samples. Nevertheless, it does not significantly affect the shape of magnetization curve, the impurity only reduces effective concentration of magnetically active species.

The preparation method is crucial for nanoparticle properties. Contrary to our findings, Priyadharshini et al.^53^ presented NiFe_2_O_4_ particles prepared by coprecipitation method using a stoichiometry ratio of 1:2 between Ni and Fe. The absence of diffraction peaks from the as-synthesized indicated an amorphous state; XRD patterns with four diffraction peaks corresponding to a cubic crystal structure were reported after annealing at 300, 400, 50, and 600 °C temperatures only. Saturation magnetization comparable to our Ni-substituted nanoparticles was found in samples annealed at 700 °C. Sol–gel autocombustion preparation^54^ of nanosized nickel ferrite led to single phase of the nickel ferrites with a spinel structure and crystallite size of 30 nm. Magnetometry revealed ferromagnetic property and saturation magnetization M_s_ between 47 and 51 Am^2^/kg. Higher value of M_s_ than that we measured in our Ni-substituted nanoparticles may reflect the nanoparticle size (30 nm compared to 9.8 nm in our case).

Magnetic resonance relaxometry revealed high *r*_2_ relaxivity of both types of nanoparticles, nevertheless, γ-Fe_2_O_3_ had higher relaxivities by approximately 20% compared to Ni_*x*_Fe_2–*x*_O_3_, see Table 3. The lower *r*_2_ relaxivity in nickel-substituted maghemite particles is obviously related to lower saturation magnetization.

**Table 3:** *r*_2_ Relaxivity of γ-Fe_2_O_3_ and Ni_*x*_Fe_2–*x*_O_3_ Nanoparticles at Room and Body Temperatures^a^

	*r*_2_ relaxivity @ 0.5 T, 23 °C (s^–1^/mM Me)	*r*_2_ relaxivity @ 0.5 T, 37 °C (s^–1^/mM Me)
γ-Fe_2_O_3_ bare	360 ± 40	297 ± 18
γ-Fe_2_O_3_ - PDMAcoAA	340 ± 40	280 ± 30
Ni_*x*_Fe_2–*x*_O_3_bare	281 ± 60	236 ± 15
Ni_*x*_Fe_2–*x*_O_3_–PDMAcoAA	320 ± 40	250 ± 40

a ‘Me’ in the units represents metallic ions (Fe in the case of γ-Fe_2_O_3_, Ni or Fe in the case of Ni_0.21_Fe_1.79_O_3_).

Uncoated ferrite nanoparticles have higher relaxivity than coated ones, which was expected, as the coating changes the distance between the core and water molecules. However, in the case of Ni_*x*_Fe_2–*x*_O_3_particles, we observed opposite behavior. We speculate that this was caused by aggregation of the uncoated particles. While mild aggregation may enhance r_2_ relaxivity, excessive aggregation or sedimentation can reduce r_2_ by decreasing the nanoparticle concentration in the major part of the measured volume. Coated particles are – due to the coating – more stable in the suspension. Interestingly, we did not observe a similar effect (aggregation of uncoated particles) in the case of γ-Fe_2_O_3_.

The lower *r*_2_ relaxivity of Ni_*x*_Fe_2–*x*_O_3_ particles may cause worse performance in MRI, however, its *r*_2_ value is still higher than that of Resovist,^3^ which was used in clinical practice particularly for liver imaging. Therefore, lower *r*_2_ does not compromise the Ni-substituted nanoparticles from use in contrast enhanced MRI (see the results of in vivo experiments). Moreover, the main goal was to investigate the substituted nanoparticles as a probe primarily for MPI, which requires quite specific nanoparticle properties, and MRI is used mainly for colocalization (MPI does not provide anatomical images).

Magnetic particle spectroscopy detected a higher signal at higher harmonics of Ni_*x*_Fe_2–*x*_O_3_ particles compared to γ-Fe_2_O_3_ (see Figure 6). In addition, coated particles revealed better performance. The better performance of nickel substituted nanoparticles was somewhat surprising. Detailed evaluation of magnetization curves and theoretical calculations did not reveal any substantial differences indicating a different response to the alternating magnetic field: relative change of the first derivation of magnetization (see Supporting Information, Figures S1 and S2) was similar for both γ-Fe_2_O_3_–PDMAcoAA and Ni_*x*_Fe_2–*x*_O_3_–PDMAcoAA particles in the range of drive fields used at MPI (see above), therefore, an effect of its lower saturation magnetization on the MPI signal is not probable.

**Figure 6:**
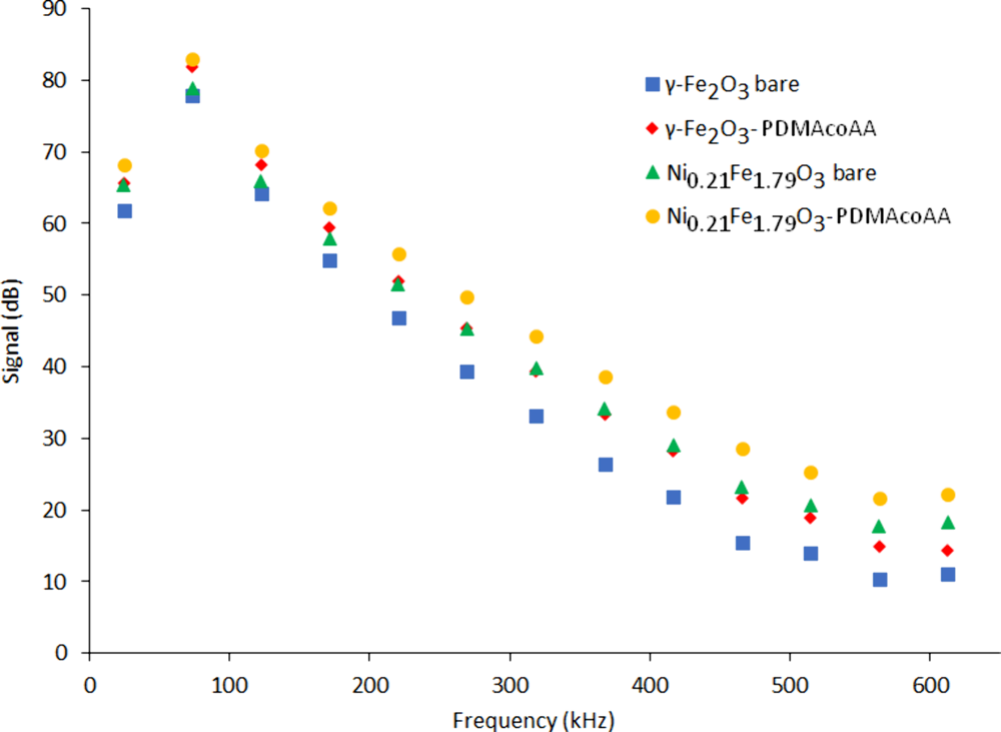
Magnetic particle spectra of γ-Fe_2_O_3_ and Ni_*x*_Fe_2–*x*_O_3_ particles obtained at an alternating magnetic field with a 25 kHz frequency and a 12 mT amplitude. Only even harmonics are shown. The low value of the first harmonics at 25 kHz is caused by the filter, which suppresses the signal originating from the excitation magnetic field.

Slight hysteresis at very low fields revealed by magnetometry in the case of γ-Fe_2_O_3_ (see Figure 5C) might cause signal ambiguity at higher frequencies in MPS. Also, amorphous-like impurities revealed in Ni-substituted nanoparticles, which reduce effective concentration of magnetically active species, may contribute to shortening of Néél relaxation time leading to a higher signal. And we should also mention small differences in the hydrodynamic size of the nanoparticles; smaller Ni_*x*_Fe_2–*x*_O_3_–PDMAcoAA (for hydrodynamic size *D*_h_ see Table 1), may exhibit a faster Brownian relaxation than γ-Fe_2_O_3_–PDMAcoAA leading to a slightly higher signal of higher harmonics (and better performance) of Ni substituted maghemite nanoparticles at frequencies around 25 kHz used in MPI.

In vitro magnetic particle imaging revealed a higher signal-to-noise ratio and a lower signal dispersion to adjacent voxels of Ni_*x*_Fe_2–*x*_O_3_ nanoparticles (both bare and PDMAcoAA-coated) compared to γ-Fe_2_O_3_ (Table 4), which confirmed the results of MPS.

**Table 4:** Signal-to-Noise Ratio of 2 × 2 × 2 mm^3^ Samples and Signal Dispersion to Adjacent Voxels Outside the Region with the Sample

	signal/noise	signal dispersion
γ-Fe_2_O_3_ bare	71 ± 25	0.038 ± 0.013
γ-Fe_2_O_3_ - PDMAcoAA	80 ± 12	0.028 ± 0.007
Ni_*x*_Fe_2–*x*_O_3_ bare	118 ± 40	0.022 ± 0.006
Ni_*x*_Fe_2–*x*_O_3_–PDMAcoAA	106 ± 23	0.022 ± 0.004

In vivo imaging confirmed that both nanoparticles are suitable for multimodal MRI/MPI imaging. The presence of nanoparticles in the mouse body was manifested by a considerable hypointense signal on MRI observable mainly in the liver immediately after their intracardial application, and in the spleen, where the minimum signal (corresponding to maximum nanoparticle concentration) was reached after 2 days (Figure 7, 8). Interestingly, the hypointense signal remained in the liver and spleen for a very long time. A significant signal increase corresponding to nanoparticle clearance from the tissue was observed 7 weeks after application in the liver and three months also in the spleen for both types of nanoparticles. Relative quantification based on MPI examinations revealed a similar course of nanoparticle content in the mouse body: a stable amount of nanoparticles was detected for 2 months and a noticeable decrease three months after nanoparticle administration (see Supporting Information, Figure S3).

**Figure 7:**
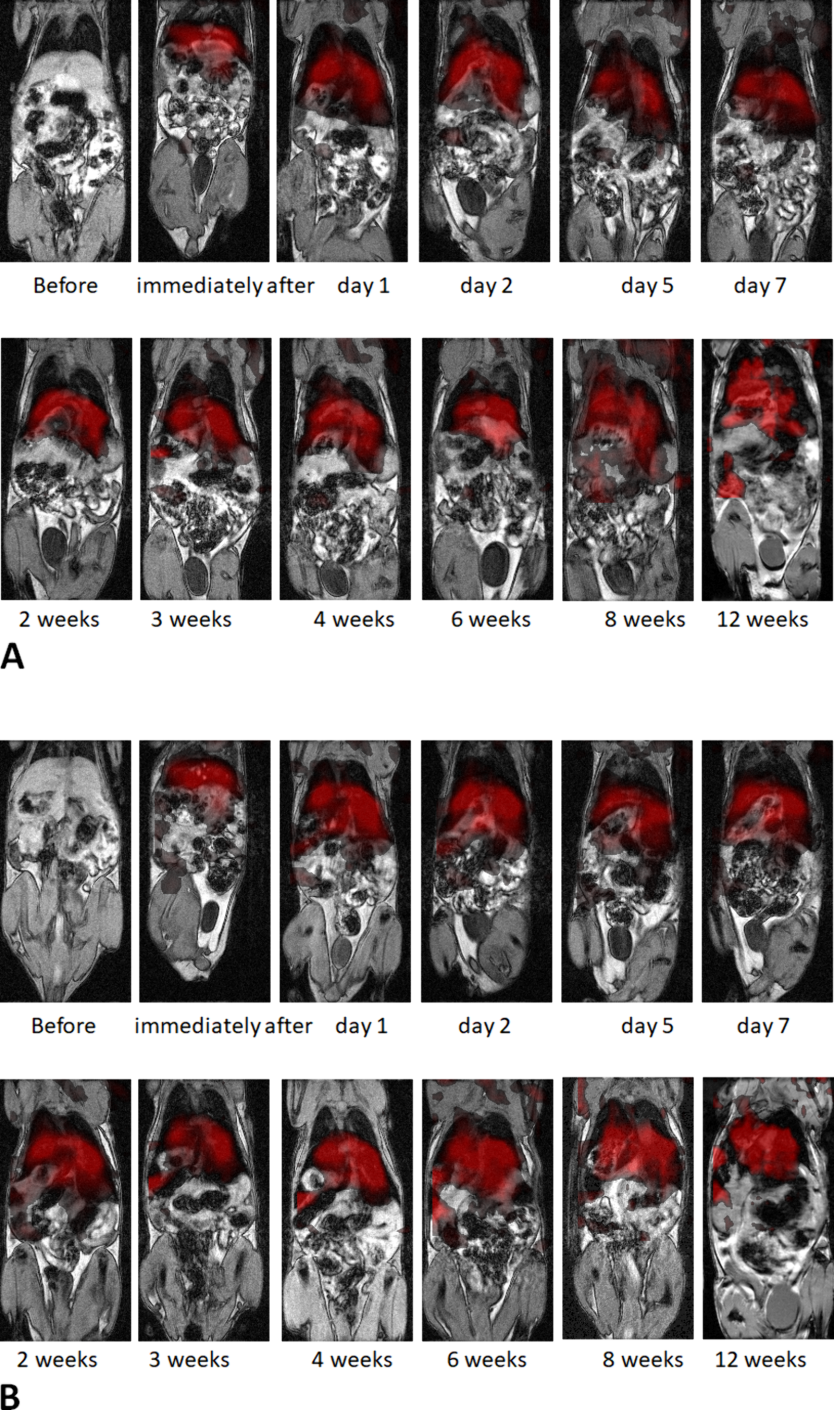
MPI (red scale) coregistered with MRI anatomical images (gray scale) at different time points. A – a mouse before and after application of γ-Fe_2_O_3_–PDMAcoAA nanoparticles; B – a mouse with Ni_*x*_Fe_2–*x*_O_3_–PDMAcoAA nanoparticles. The results of MPI multipatch reconstruction (2 patches in the *x*-direction) are presented.

**Figure 8:**
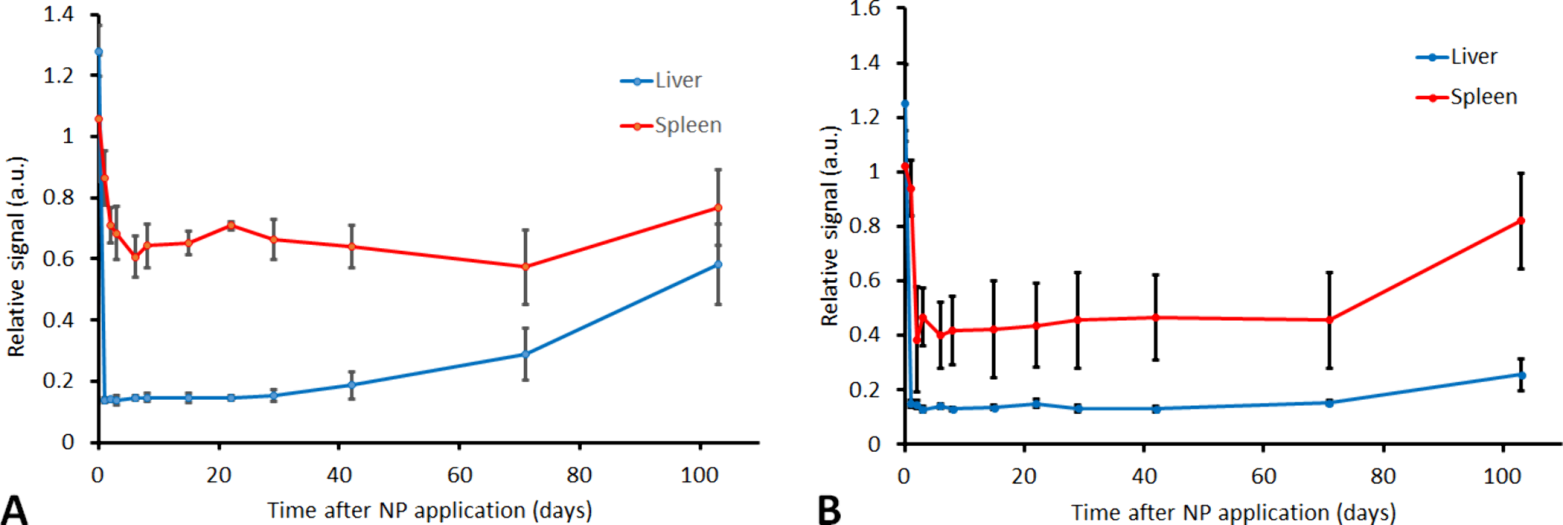
Evolution of a relative MRI signal in the liver and spleen in vivo after intracardial application of γ-Fe_2_O_3_– PDMAcoAA (A) and Ni_*x*_Fe_2–*x*_O_3_–PDMAcoAA nanoparticles (B).

Similarly, MPI detected particles in the liver and spleen (Figure 6). During long-term follow-up measurements, the MPI signal was also found in the lymph nodes in the case of several animals. No substantial differences were observed between the performance of γ-Fe_2_O_3_–PDMAcoAA and Ni_*x*_Fe_2–*x*_O_3_–PDMAcoAA nanoparticles in vivo. In one animal, MPI also detected nanoparticles in the heart. As it was observed in one animal only, we presume that the reason was an imperfect intracardial application, during which part of the nanoparticles was deposited in the myocardial muscle; see Supporting Information, Figure S4.

The long retention of the nanoparticles in the tissue is surprising (Figure 8). Iron oxide nanoparticles (Resovist) were also used in clinical practice and were believed to be cleared from the human body within one or 2 weeks.^55,56^ However, long retention of iron oxide nanoparticles after application to mice was also described by Charvatova et al.^57^ Resovist clearance in mice was very slow and its half-life was calculated to be 290 days, indicating a substantial difference in the clearance of iron oxide nanoparticles between humans and mice used as an experimental model. The dose was calculated according to Nair et al.,^58^ which considered not only animal body weight, but also the body surface, to match an equivalent dose used in humans; however, with respect to the results, it is questionable whether the extrapolation based on the body area does not overestimate the calculated dose with regard to liver clearance capacity. Our observation indicates that literature-recommended doses may need adjustment in future preclinical studies.

The short-term distribution of Ni_*x*_Fe_2–*x*_O_3_–PDMAcoAA was also confirmed by mass spectrometry. *Post-mortem* evaluation of nickel content in various organs 24 h after application showed a high concentration of Ni in the liver and spleen, a negligible concentration was found in other organs examined (kidney, lung, lymph nodes), which is in line with the imaging (Figure 9). As nickel is only found in trace amounts in the body under normal conditions, we may presume that most of the detected metal had its origin in Ni_*x*_Fe_2–*x*_O_3_–PDMAcoAA nanoparticles. Unfortunately, mass spectrometry did not allow the detection of γ-Fe_2_O_3_–PDMAcoAA nanoparticles, as it is not capable of distinguishing natively present iron (for example, heme iron) from iron originating in nanoparticles.

**Figure 9:**
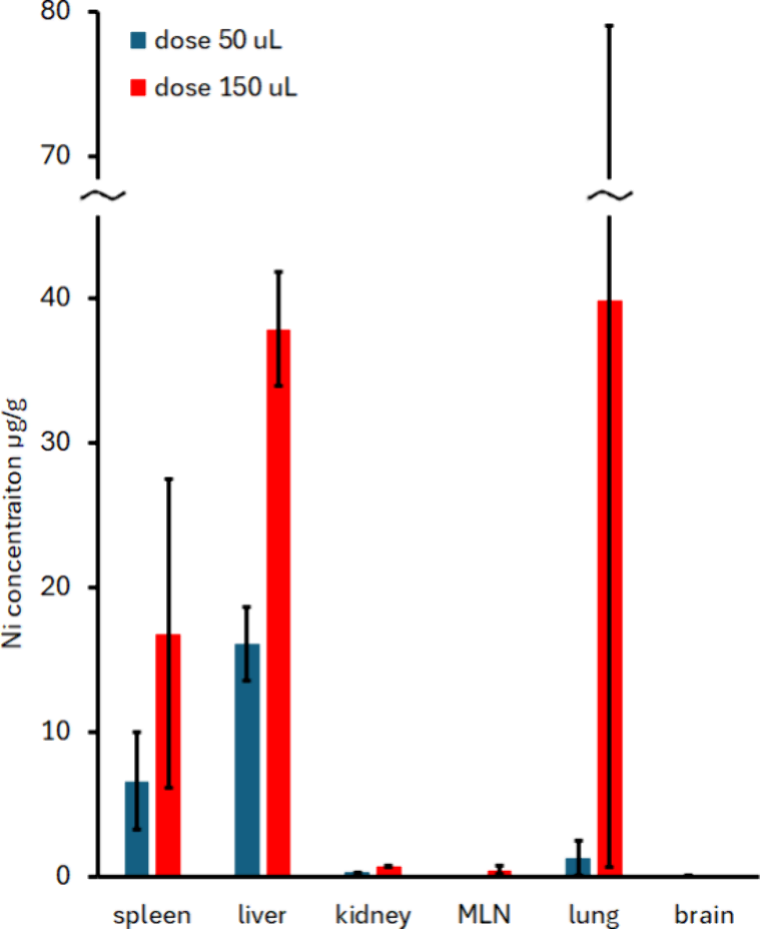
Quantitation of nickel content 24 h after intracardial application of Ni_*x*_Fe_2–*x*_O_3_–PDMAcoAA particles. The amount of nickel in a control animal was below the detection limit in all organs evaluated.

The results corresponded to MRI and MPI findings: high entrapment of nanoparticles in the liver immediately after application, slower nanoparticle deposition in the spleen, and almost no presence of nanoparticles in the kidney or lungs.

We did not observe any significant difference in the detection of γ-Fe_2_O_3_–PDMAcoAA or Ni_*x*_Fe_2–*x*_O_3_–PDMAcoAA in vivo. In general, in vivo measurements suffer from higher noise, which may cover small differences in particle performance. Also, if the differences in MPI signal reflect a different Brownian relaxation in water suspensions in vitro, they could be effectively effaced by mixing nanoparticles with blood and blood elements (namely proteins), which probably create a larger corona around the nanoparticles and prolong the Brownian relaxation. Finally, image reconstruction using the system function and averaging when multipatch measurement is used may also erase minor differences in signal-to-noise ratio.

While MPI provides a highly specific signal, there are several substantial drawbacks of this imaging method. The essential problem is a small field of view. In our settings, the basic FOV was 24 mm (length) × 24 mm (width) × 12 mm (height). This can be enlarged using the focus fields, when the small FOV (a ‘patch’) is shifted in different directions and individual FOVs (with overlap) are then merged.^59^ Merging is complicated by the so-called border artifacts, which may cause a high signal on the borders of the patches. Simple averaging of overlapping areas resulted in periodical areas with an artificially higher signal.^60^ Several methods were proposed to suppress these artifacts.^61^ In this study, we used a Gaussian weighted averaging with zero weight on the border slices to suppress signals from the patch borders. Even in the case of weighted averaging, we experienced in several experiments a strong influence of the border artifacts (see Supporting Information, Figure S5), when we used a higher number of patches (18) for image reconstruction. Therefore, we suppose that attention should be paid to the number of patches and limit them to the lowest possible number, while keeping a sufficient overlap of patches and using weighted averaging. In our case, we obtained the best results when we used only two patches and 50% overlap, which covered volume 36 mm (length) × 24 mm (width) × 12 mm (height). This FOV was sufficient to image most of the mouse body (chest + abdomen).

The dimethylacrylamide (PDMAcoAA) used for the coating has already been successfully tested for various biomedical applications;^31,62–64^ it ensures stability of the coated nanoparticles and their safety for use in vivo. Moreover, it can be further functionalized for specific applications.

## Conclusions

This work aimed to synthesize and characterize nanoparticles with improved properties compared to standard superparamagnetic iron oxide-based nanoparticles for use in magnetic particle imaging, achieved by substituting iron ions in the core with nickel. The Ni-substituted particles were characterized using TEM, XRD, Mössbauer spectroscopy, magnetometry, and were compared to γ-Fe_2_O_3_. Nickel substitution resulted in superparamagnetic Ni_*x*_Fe_2–*x*_O_3_ nanoparticles, which exhibited a higher magnetic particle spectroscopy signal and superior MPI performance. Although their lower saturation magnetization leads to reduced *r*_2_ relaxivity—and thus potentially slightly decreased MRI performance (used primarily for colocalization) —the relaxivity remains sufficient to provide excellent MRI contrast. Importantly, both types of nanoparticles (γ-Fe_2_O_3_ and Ni_*x*_Fe_2–*x*_O_3_) proved suitable for combined MRI/MPI imaging. MPI complements anatomical MRI images by offering a highly specific signal of the tracer.

In vivo experiments confirmed that upon administration, the nanoparticles are rapidly entrapped in the liver, where they are readily detected by both MRI (as a nonspecific hypointense signal) and MPI (as a highly specific signal). Within one or 2 days, the nanoparticles also accumulate in the spleen, while MPI detects their presence in lymph nodes over an extended period. The dimethylacrylamide-based coating ensures nanoparticle stability and biocompatibility and offers potential for future functionalization tailored to specific biomedical applications.

## Supplementary Material



## Notes

Additional details supporting the discussion of magnetization behavior (Figures S1,2), nanoparticle quantification in vivo (Figure S3), anomalous distribution of nanoparticles (Figure S4), and border artifacts (Figure S5), and zero-field Mössbauer spectra (Figure S6) (PDF)
