# Supplementary material for: Nickel Ferrite Nanoparticles for In Vivo Multimodal Magnetic Resonance and Magnetic Particle Imaging
Source: ACS Appl Nano Mater. 2025 Jul 16;8(29):14867–81. doi: 10.1021/acsanm.5c03013 (PMC12308752; doi:10.1021/acsanm.5c03013)
Supplement: Supplementary file 1 [file an5c03013_si_001.pdf]

Supporting Information

Nickel Ferrite Nanoparticles for *in vivo* Multimodal Magnetic Resonance and Magnetic Particle Imaging

Vít Herynek[1], Lenka Rajsiglová[2,3], Michal Babič[4], Monika Švábová[4], Jaroslav Kohout[5*], Miroslav Veverka[5], Tomáš Kmječ[5], Lenka Kubíčková[6], Jiří Karela[1], Filip Gregar[7], Martin Loula[8], Stanislava Matějková[8], Luděk Šefc[1], Luca Vannucci[2]*

[1] Center for Advanced Preclinical Imaging (CAPI), First Faculty of Medicine, Charles University, Prague, Czech Republic
[2] Laboratory of Immunotherapy, Institute of Microbiology, Czech Academy of Sciences, Prague, Czech Republic
[3] Department of Cell Biology, Faculty of Science, Charles University, Prague, Czech Republic
[4] Polymer and Colloid Immunotherapeutics, Institute of Macromolecular Chemistry, Czech Academy of Sciences, Prague, Czech Republic
[5] Department of Low Temperature Physics, Faculty of Mathematics and Physics, Charles University, Prague, Czech Republic
[6] Department of Magnetics and Superconductors, Institute of Physics, Czech Academy of Sciences, Prague, Czech Republic
[7] Department of Analytical Chemistry, Faculty of Science, Palacký University Olomouc, Olomouc, Czech Republic
[8] Analytical Laboratory, Institute of Organic Chemistry and Biochemistry, Prague, Czech Academy of Sciences, Czech Republic

Corresponding Authors:
MSc. Vít Herynek, Ph.D., Center for Advanced Preclinical Imaging (CAPI),
First Faculty of Medicine, Charles University, Salmovská 3, 120 00, Prague, Czech Republic
E-mail: vit.herynek@lf1.cuni.cz
Office phone: +420 224 965 993

Ass. Prof. MSc. Jaroslav Kohout, Ph.D., Department of Low Temperature Physics,
Faculty of Mathematics and Physics, Charles University
V Holešovičkách 2, 180 00, Prague, Czech Republic
e-mail: jaroslav.kohout@mff.cuni.cz
phone: +420 951 552 771

Magnetization and MPI Signal

The MPI signal is generated by nanoparticles exposed to a sinusoidally varying magnetic field, called drive field. Their magnetization oscillates with the same frequency. Neglecting relaxation mechanisms, the magnetization follows sinusoidal behavior at low amplitudes. At higher amplitudes, the magnetization, which response to an external magnetic field is given by the Langevin function, is no longer sinusoidal, and the signal is described by the following equation:

$$S(t) \approx \frac{dM}{dt} = N\,\mu \frac{d}{dt}\,L\left(\frac{\mu}{kT}\,B\sin(\omega t)\right), \qquad \text{(S1)}$$

where t is time, M is magnetization, μ is permeability, L is Langevin function, B is the amplitude of the excitation magnetic field, and ω is the angular frequency of the excitation field.

Due to its non-sinusoidal behavior, the signal contains higher harmonics, which are crucial for signal detection and processing, as the first harmonic needs to be filtered out to remove a contribution of the excitation drive field to the signal. Therefore, it is necessary that the amplitude of the excitation magnetic field is high enough to reach the nonlinear part of the Langevin function, i.e., area, where the first derivative of the magnetization substantially differs from a constant value.

We fitted the magnetization curves and calculated its first derivative in the range of relevant drive field amplitudes (i.e., ± 15 mT), see Fig. S1. The relative change of the derivate is for both types of nanoparticles (γ-Fe_2O_3-PDMAcoAA and γ-$Ni_xFe_{2-x}O_3$-PDMAcoAA) within the experimental error and the fitting error the same, which implies no differences in the MPI signal. The simulation of the signal induced by sinusoidal excitation after deduction of the sinusoidal component (corresponding to the first harmonic) is shown in Fig. S2; it revealed just negligible differences between the signals of the two types of nanoparticles and confirmed the conclusion based on comparison of the first derivatives.

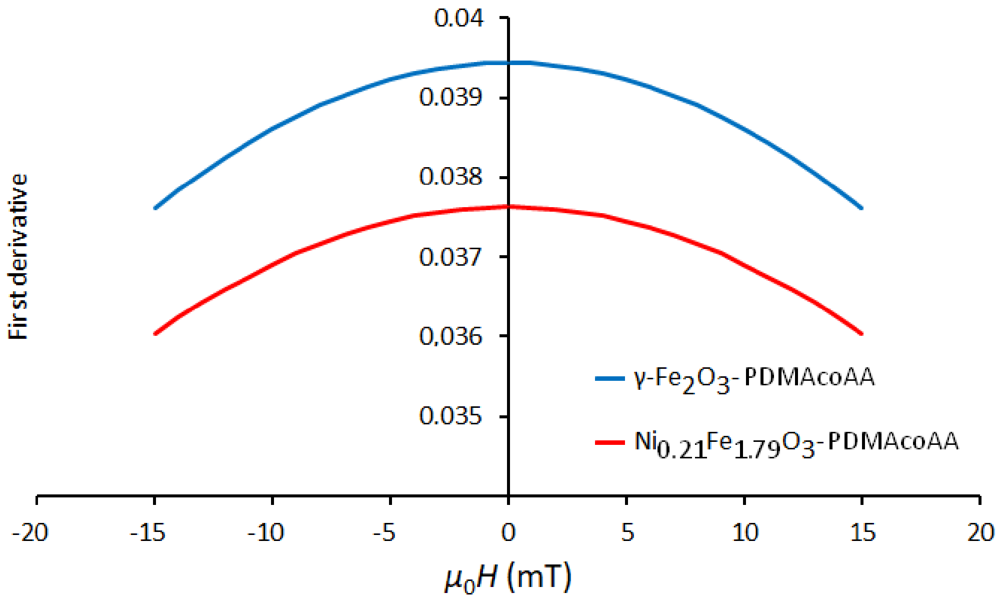

Fig. S1: The first derivative of the magnetization curve of γ-Fe_2O_3-PDMAcoAA and $Ni_xFe_{2-x}O_3$-PDMAcoAA nanoparticles. The magnetization curve was fitted by a Langevin function (Eq. S1) prior to derivation.

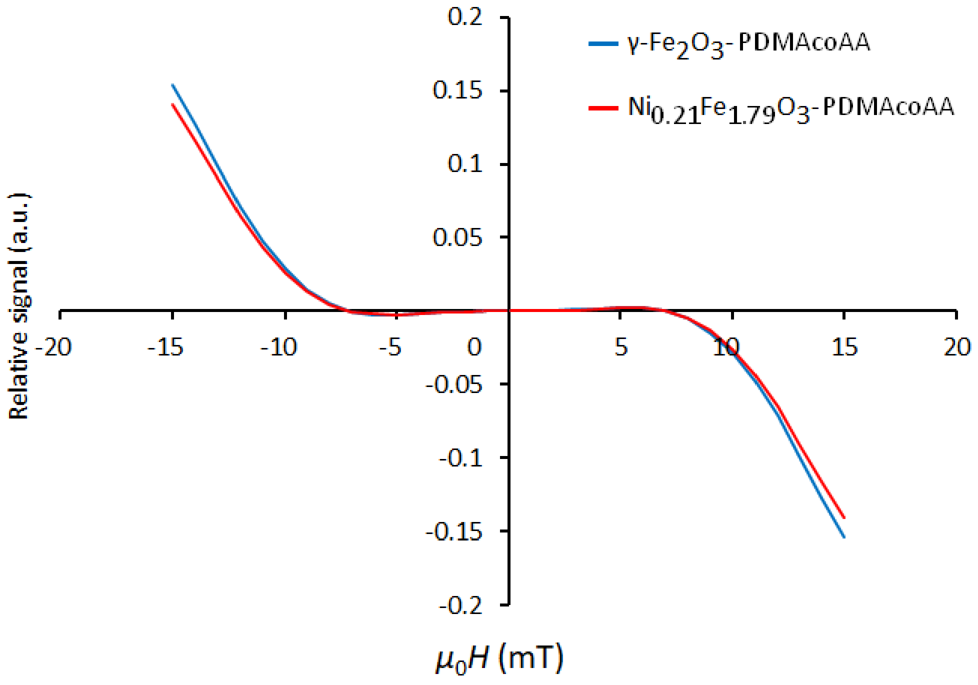

Fig. S2: The theoretical signal generated by γ-Fe_2O_3-PDMAcoAA and γ-$Ni_xFe_{2-x}O_3$-PDMAcoAA nanoparticles. The first harmonic (i.e., sinusoidal signal at the frequency of the excitation drive field) is deducted.

Quantitation of MPI Signals in *In Vivo* Examinations

MPI provides a quantifiable signal. Absolute quantification can be performed with an external phantom with known amount of the tracer used. We did not use an external phantom, however, a relative quantification of total nanoparticle content in the mouse body was performed. A total signal was calculated at each time point; Fig. S4 shows average values across the two experimental groups (a group with administered γ-Fe_2O_3-PDMAcoAA nanoparticles and a group with administered $Ni_xFe_{2-x}O_3$-PDMAcoAA nanoparticles. The results correspond to MRI examination: the nanoparticle content was roughly stable for 60 days, then it started to decrease.

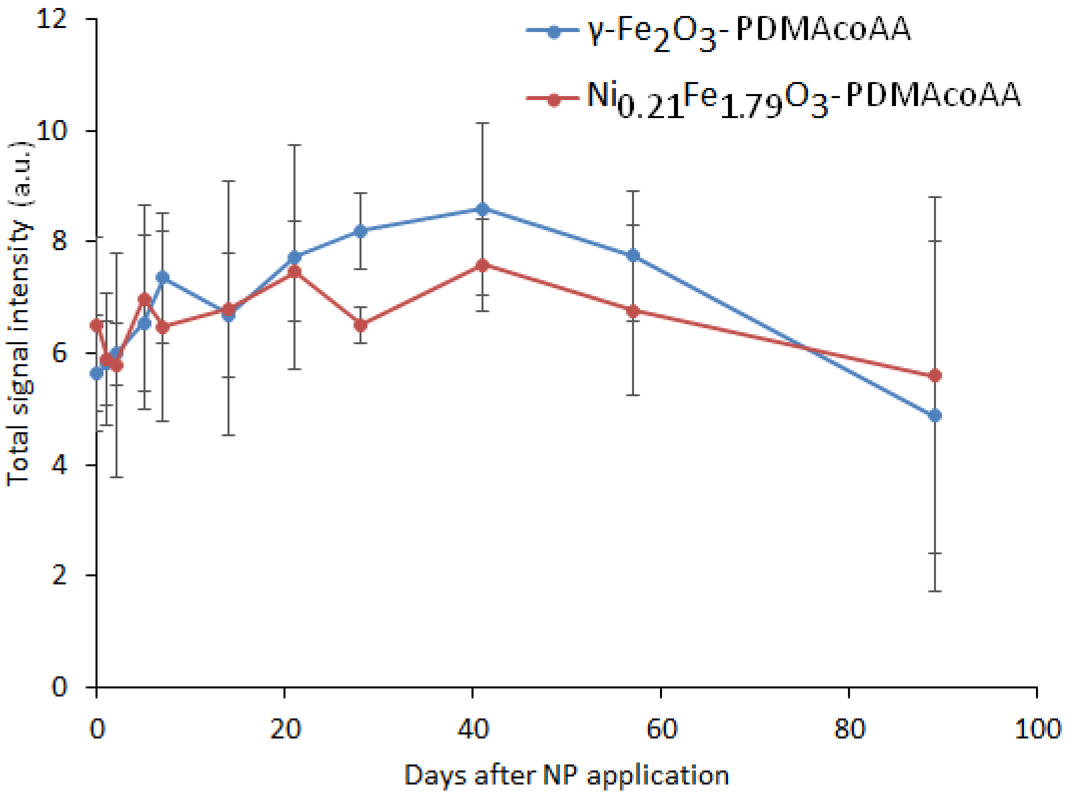

Fig. S3: Relative content of nanoparticles in the mouse body after intracardial administration of γ-Fe_2O_3-PDMAcoAA or $Ni_xFe_{2-x}O_3$-PDMAcoAA nanoparticles.

Intracardial Nanoparticle Administration

The *in vivo* experiment included intracardial administration of nanoparticles. It is a simple and minimally invasive procedure; the needle should be inserted through the myocardium into a cardiac chamber. The nanoparticles are then rapidly distributed by the bloodstream to the whole body. The administration was successful in all animals, although we noticed imperfect administration in one animal, when a certain amount of the nanoparticles was deposited also in the myocardial muscle during the perforation of the heart wall. This was nicely documented by MPI imaging. For a comparison of animals with and without a nanoparticle deposition in the myocardium during administration, see Fig. S4.

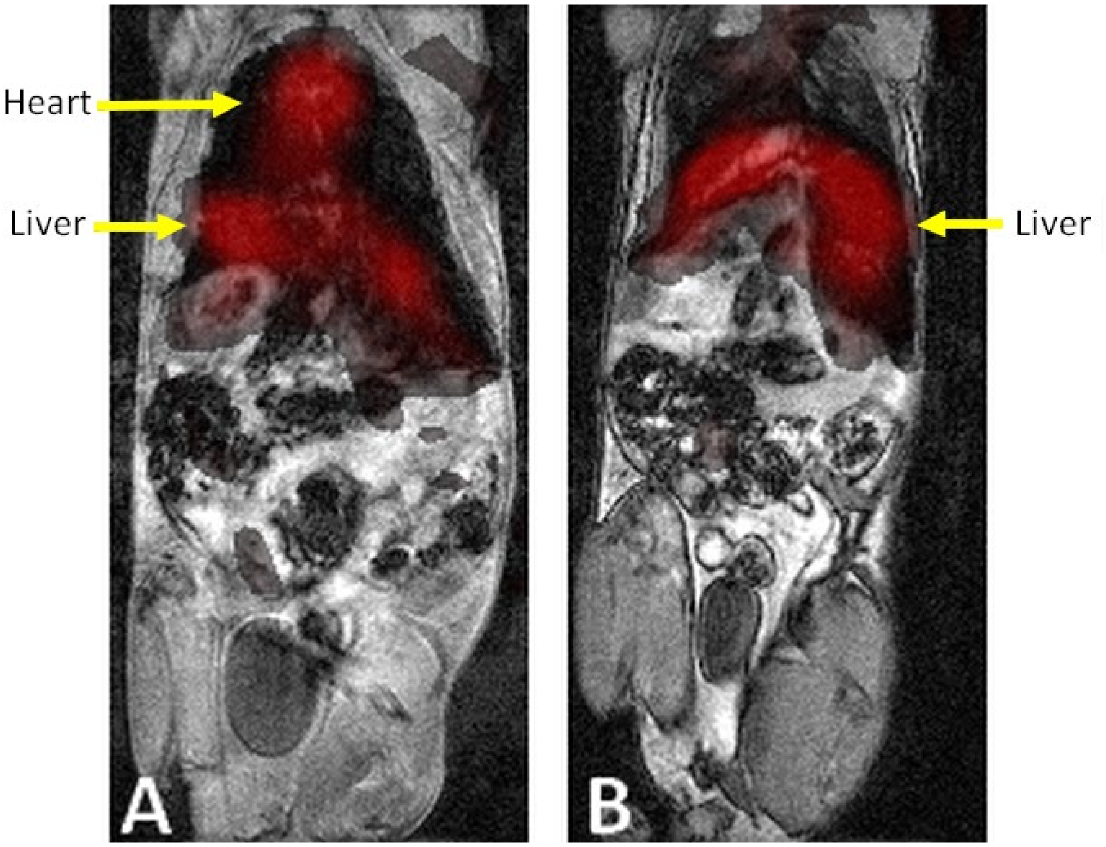

Fig. S4. MRI and MPI images of animals scanned immediately after the administration of γ-Fe_2O_3-PDMAcoAA nanoparticles. A – an animal with partial deposition of the nanoparticles in the myocardium; B – an animal with correctly injected nanoparticles in the blood stream.

Border Artifacts in MPI

Multipatch MPI scanning solves a problem with a small field of view (FOV) by merging individual small FOVs (‘patches‘) together. The method is complicated by the so-called border artifacts, which may cause an artificial high signal on the borders of the patches. Simple averaging of overlapping areas may result in periodical areas with higher signal, and weighted averaging is necessary. Even in the case of weighted averaging, we sometimes experienced strong influence of the border artifacts, when we used a higher number of patches (e.g., 18) for image reconstruction. Therefore, we suppose that the number of patches should be limited to the lowest possible number, while keeping a sufficient overlap of patches and using weighted averaging. An extreme case of border artifacts is documented in Fig. S5.

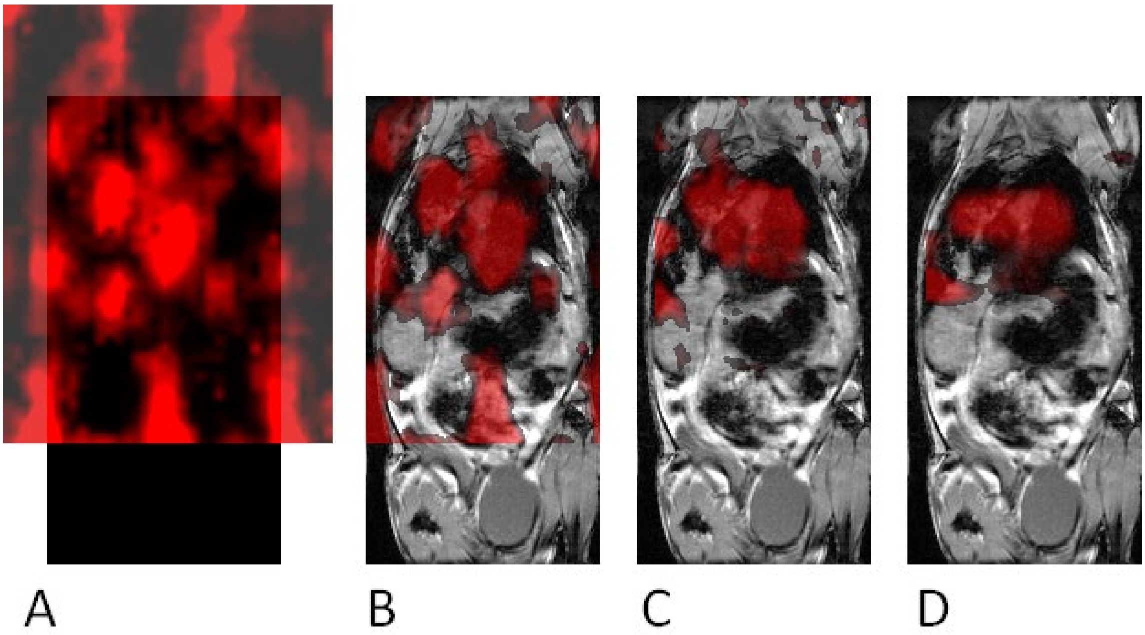

Fig. S5: Demonstration of border artifacts after multipatch reconstruction of 18 patches.

A - MPI multipatch image with strong artifacts obtained by combination of 18 patches; an area overlapping with the corresponding MRI field of view is highlighted; B – colocalization of the same image with MRI; C - MPI multipatch image obtained using 2 patches and colocalized with MRI; D - single patch colocalized with MRI.

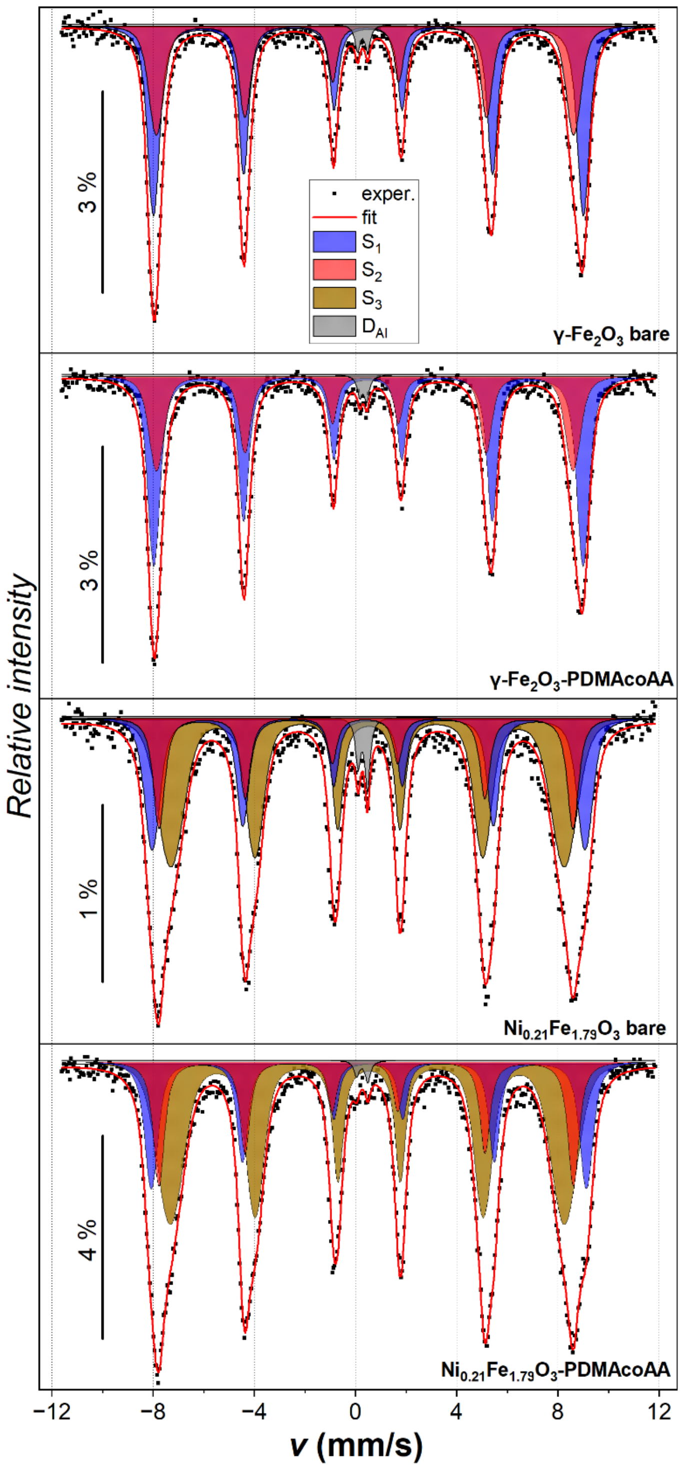

Figure S6: The zero-field Mössbauer spectra shows an asymmetric sextet, whose decomposition into two magnetic components, attributed to the tetrahedral and octahedral Fe sites, remained ambiguous.

Table S1 - Hyperfine parameters determined from the ^{57}Fe Mössbauer spectra of the bare and PDMAcoAA-functionalized γ-Fe_2O_3 and Ni-substituted nanoparticles at 4.2 K. Parameter designations: B_{ext} – external magnetic field, *IS* – isomer shift, *QS* – quadrupole shift (splitting for D_{Al}), B_{eff} – mean value of the effective magnetic field on ^{57}Fe nuclei $B_{eff} = |\mathbf{B}_{hf} + \mathbf{B}_{ext}|$, *I* – integral intensity. Numbers in parentheses represent the standard deviation of the last digit.

Sample	B_{ext} [T]	Comp.	Sites	*IS* [mm/s]	*QS* [mm/s]	B_{eff} [T]	*I* [%]	*b*
γ-Fe_2O_3 bare	0	S_1	Octahedral (Fe^{3+})	0.50(4)	0.01(3)	$52.7(3)^+$	63*	2.06(3)
		S_2	Tetrahedral (Fe^{3+})	0.38(3)	-0.04(3)	$51.1(3)^+$	35*	2.06(3)
		D_{Al}	Al foil (0.5%Fe)	0.28(4)	0.40(5)	-	2(1)	-
	6	S_1	Octahedral (Fe^{3+})	0.50(2)	0.00(3)	47.1(3)	63(1)	3.65(3)
		S_2	Tetrahedral (Fe^{3+})	0.39(3)	0.01(3)	57.5(3)	35(1)	3.65(5)
		S_{Al}	Al foil (0.5%Fe)	0.36(5)	0.41(6)	2.9(5)	2(1)	4*
γ-Fe_2O_3-PDMAcoAA	0	S_1	Octahedral (Fe^{3+})	0.50(4)	0.02(3)	$52.7(3)^+$	63*	2.03(3)
		S_2	Tetrahedral (Fe^{3+})	0.37(3)	-0.03(3)	$51.1(3)^+$	35*	2.03(3)
		D_{Al}	Al foil (0.5%Fe)	0.30(5)	0.29(6)	-	2(1)	-
	6	S_1	Octahedral (Fe^{3+})	0.51(2)	0.01(3)	47.1(2)	63(1)	3.75(3)
		S_2	Tetrahedral (Fe^{3+})	0.38(3)	-0.02(3)	57.5(3)	35(1)	3.75(5)
		S_{Al}	Al foil (0.5%Fe)	0.30(5)	0.40(6)	2.7(5)	2(1)	4*
$Ni_xFe_{2-x}O_3$ bare	0	S_1	Octahedral (Fe^{3+})	0.51(3)	0.03(3)	$53.5(3)^+$	22(1)	2.03(3)
		S_2	Tetrahedral (Fe^{3+})	0.41(3)	0.04(4)	$51.0(3)^+$	22(1)	2.03(3)
		S_3	FeOOH (Fe^{3+})	0.49(3)	-0.05(3)	$48.6(3)^+$	54(2)	2.03(3)
		D_{Al}	Al foil (0.5%Fe)	0.28(3)	0.37(4)	-	2(1)	-
	6	S_1	Octahedral (Fe^{3+})	0.50(3)	0.04(4)	47.7(3)	22(3)	3.6(1)
		S_2	Tetrahedral (Fe^{3+})	0.40(3)	0.01(3)	56.9(3)	22(3)	3.6(1)
		S_3	FeOOH (Fe^{3+})	0.51(3)	-0.06(4)	47.6(3)	55(5)	2.6(1)
		S_{Al}	Al foil (0.5%Fe)	0.26*	-	2.2(4)	2(1)	4*
$Ni_xFe_{2-x}O_3$-PDMAcoAA	0	S_1	Octahedral (Fe^{3+})	0.52(3)	0.02(3)	$53.5(3)^+$	22(2)	2.07(3)
		S_2	Tetrahedral (Fe^{3+})	0.40(3)	0.05(3)	$51.0(3)^+$	22(2)	2.07(3)
		S_3	FeOOH (Fe^{3+})	0.50(3)	-0.07(3)	$48.6(4)^+$	56(4)	2.07(3)
		D_{Al}	Al foil (0.5%Fe)	0.26(5)	0,44(7)	-	1(1)	-
	6	S_1	Octahedral (Fe^{3+})	0.51(2)	0.06(4)	47.6(3)	22(1)	3.3(1)
		S_2	Tetrahedral (Fe^{3+})	0.38(3)	0.01(3)	56.9(3)	22(1)	3.3(1)
		S_3	FeOOH (Fe^{3+})	0.49(3)	-0.09(4)	47.4(3)	56(2)	2.6(1)
		S_{Al}	Al foil (0.5%Fe)	0.29(9)	-	1.1(7)	1(1)	4*

* Fixed, $^+ B_{hf} = B_{eff}$
